# Prepubertal Ovariectomy Exaggerates Adult Affective Behaviors and Alters the Hippocampal Transcriptome in a Genetic Rat Model of Depression

**DOI:** 10.3389/fendo.2017.00373

**Published:** 2018-01-22

**Authors:** Neha S. Raghavan, Hao Chen, Matthew Schipma, Wendy Luo, Sarah Chung, Lei Wang, Eva E. Redei

**Affiliations:** ^1^The Asher Center for the Study & Treatment of Depressive Disorders, Feinberg School of Medicine, Northwestern University, Chicago, IL, United States; ^2^Department of Psychiatry and Behavioral Sciences, Feinberg School of Medicine, Northwestern University, Chicago, IL, United States; ^3^Department of Pharmacology, The University of Tennessee Health Science Center, Memphis, TN, United States; ^4^Next-Generation Sequencing Core Facility, Northwestern University, Chicago, IL, United States; ^5^Department of Radiology, Feinberg School of Medicine, Northwestern University, Chicago, IL, United States

**Keywords:** depression-like behavior, Wistar Kyoto more immobile, forced swim test, RNA-Seq, differentially expressed genes

## Abstract

Major depressive disorder (MDD) is a debilitating illness that affects twice as many women than men postpuberty. This female bias is thought to be caused by greater heritability of MDD in women and increased vulnerability induced by female sex hormones. We tested this hypothesis by removing the ovaries from prepubertal Wistar Kyoto (WKY) more immobile (WMI) females, a genetic animal model of depression, and its genetically close control, the WKY less immobile (WLI). In adulthood, prepubertally ovariectomized (PrePubOVX) animals and their Sham-operated controls were tested for depression- and anxiety-like behaviors, using the routinely employed forced swim and open field tests, respectively, and RNA-sequencing was performed on their hippocampal RNA. Our results confirmed that the behavioral and hippocampal expression changes that occur after prepubertal ovariectomy are the consequences of an interaction between genetic predisposition to depressive behavior and ovarian hormone-regulated processes. Lack of ovarian hormones during and after puberty in the WLIs led to increased depression-like behavior. In WMIs, both depression- and anxiety-like behaviors worsened by prepubertal ovariectomy. The unbiased exploration of the hippocampal transcriptome identified sets of differentially expressed genes (DEGs) between the strains and treatment groups. The relatively small number of hippocampal DEGs resulting from the genetic differences between the strains confirmed the genetic relatedness of these strains. Nevertheless, the differences in DEGs between the strains in response to prepubertal ovariectomy identified different molecular processes, including the importance of glucocorticoid receptor-mediated mechanisms, that may be causative of the increased depression-like behavior in the presence or absence of genetic predisposition. This study contributes to the understanding of hormonal maturation-induced changes in affective behaviors and the hippocampal transcriptome as it relates to genetic predisposition to depression.

## Introduction

Major depressive disorder (MDD) is a devastating and prevalent disorder. Pubertal development is a major factor in the first onset of MDD, particularly in females. While ~7% of 13–14 year olds are affected, this percentage doubles in 17–18 year olds (1). During preadolescence, the prevalence of MDD shows no gender difference, but after puberty and until menopause, the incidence of depression is twice as high in women as in men (1). Anxiety, which is frequently comorbid with MDD, is also more common in female MDD (2).

In addition to the many social, physiological, and psychological changes that occur during female puberty that partially explain the increased incidence of MDD in adolescence, increased and cyclic secretion of estradiol (E2) and progesterone are proposed to contribute to the risk. One mechanism by which this may occur is through E2’s ability to regulate the stress-responsive hypothalamic–pituitary–adrenal axis (3, 4), which is known to be closely involved in the etiology of MDD. During periods of life when large changes in levels of E2 occur, vulnerability for the incidence of depression and anxiety also increases (5, 6). Studies have demonstrated that heightened anxiety tends to occur at times when E2 is lower, such as premenstrually and postpartum, while women report decreased anxiety during periods of higher E2 levels (7), suggesting an anxiolytic role of E2. Animal studies mirror these E2-associated antianxiety effects (8, 9), manifested in decreased anxiety-like behavior from adolescence to adulthood (10–12), and increased anxiety-like behavior after prepubertal ovariectomy (PrePubOVX) (13). In adulthood, both low levels of estrogen and progesterone and the complete lack of these hormones after postpubertal ovariectomy have been associated with greater occurrence of depression-like behavior (14–16). Thus, a paradox clearly exists between the increased incidence of MDD and anxiety after puberty in women, and the anxiolytic and antidepressant roles of E2.

The study of the interaction of genetics and hormones in MDD has proved even more elusive as genetic risks contributing to MDD have just begun to be identified (17). As shown by both human and animal studies, partially overlapping, but mostly distinct, genetic and/or epigenetic mechanisms precipitate MDD in males and females (18, 19). In particular, women tend to have a higher heritability for the disorder than men (20), and the genetic architecture of depression-like behavior is sexually dimorphic (19). Previously, we have shown that while male Wistar Kyoto (WKY) more immobile (WMI) inbred rats display depression-like behavior before and after puberty, females exhibit increased depression-like behavior only after puberty (21), paralleling the higher prevalence of MDD in postpubertal women compared with men.

The WMIs are derived from the near-inbred WKY rat strain, an established model of major depression with comorbid anxiety in adulthood (19, 22–26) and adolescence (27, 28). Two inbred strains were generated from the WKYs by selective breeding using the immobility behavior in the forced swim test (FST) as a functional selector. These are the WMI showing despair-like behavior and its genetically similar, but behaviorally different control strain, the WKY less immobile (WLI). Both depression- and anxiety-like behaviors increase between early adolescence and adulthood in the female WMIs (21). These strains provide a unique opportunity to begin exploring the role of puberty in the development of depression- and anxiety-like behaviors in females with or without stable genetic predisposition.

Further, gene expression differences in the hippocampus between the WMIs and WLIs have been previously reported (21, 29, 30). The hippocampus is known to show abnormalities in mood disorders (31, 32) and undergo significant remodeling during the pubertal period (33). In both humans (34, 35) with depression and rodent models of depression (36, 37), alterations in both volumetric and functional connectivity have been reported. A previous microarray study of the WLI and WMI males found significant, large (higher than 3×) expression differences between the strains in the hippocampus (29). Of the most significant differentially expressed genes (DEGs), strain differences have also been found between early adolescent (EA) and adult WMI and WLI females (21). For these reasons, although multiple other regions are involved in the pathophysiology of depression, we chose to focus on the hippocampus for this study.

This study investigated the consequences of ovariectomy before puberty on the adult WMI’s and WLI’s behavior in the FST and in the open field tests (OFTs), a routinely used test of anxiety-like behavior. Transcriptome differences by strain and treatment were examined by RNA-Seq in the hippocampus. In addition to global transcriptome changes in the hippocampus, we focused on changes in transcript levels of the estrogen receptors, which are present in high density in the hippocampus (7) and glucocorticoid receptors, known to be involved in mood and depression-like behaviors (38). We hypothesized that prepubertal ovariectomy affects both the behavior and gene expression of WMIs and WLIs differently.

## Materials and Methods

### Animals

The Institutional Animal Care and Use Committee of Northwestern University approved all animal procedures. The WMI and WLI strains have been maintained in our vivarium with continuous brother–sister mating throughout the past 35 generations (21, 39). Animals were housed under temperature and humidity control, a 14:10 h light–dark cycle, with lights on at 0600 hours. Food and water were available *ad libitum*.

Prepubertal WMI and WLI female rats, 26–28 days old, were anesthetized using a ketamine–xylazine cocktail (4 mL/kg containing 10 mg ketamine and 1.25 mg xylazine in 0.9 mL H_2_O) and either underwent bilateral ovariectomy (PrePubOVX, *N* = 12 WMI, 17 WLI) or Sham operation (Sham, *N* = 11 WMI, 15 WLI). Differences in animal numbers between the strains are due to the lower fecundity of WMIs compared with the WLIs. The timing of ovariectomy was chosen as to preclude pubertal changes, which can start at postnatal day (PN) 28, but generally begin from PN 33 onward (40). Figure S1 in Supplementary Material shows the detailed experimental design. Intact WMI and WLI females were also employed in the FST to determine if Sham surgery before puberty cause any change in this behavior, which was the original selector of these strains. We expected that the differences between Sham and PrePubOVX measures would be a magnitude higher than estrus cycle-related differences, and therefore we did not control for estrus cycle phases of the Sham or intact females.

To compare the effect of the lack of ovarian hormones between early adolescence (before puberty) and the adult state after PrePubOVX on the hippocampal expression of the estrogen and glucocorticoid receptors, we included EA animals in those specific studies.

### Behavioral Testing

Behavioral testing began at approximately postnatal day 90 with the OFT as described previously (21). Briefly, animals were placed in the center of an 82-cm diameter arena, with an internal, central illumination of 77 lux. The test lasted 10 min, and the number of times the animal entered into the center 50-cm diameter area and the % time spent there were measured by TSE Videomot 2 version 5.75 software (Technical & Scientific Equipment, Bad Homburg, Germany). Anxious animals exhibit increased thigmotaxis and spend less time in the center of the open arena.

Two days later, FST was performed (prior OFT does not affect FST behavior) as described previously (19). Briefly, on the first day of the 2-day test, adult animals were placed in a tank filled with 22–24°C water for 15 min. On day 2, animals were again placed in the tank for a 5-min session, which was videotaped and later scored by a trained observer, blind to the strain or the treatment of the animal. Intact same age animals from a different generation were also tested in the FST to determine the effect of Sham surgery on this behavior. It is thought that increased time spent immobile in the FST indicates increased despair-like behavior.

### RNA Isolation

Five weeks after behavioral testing, PrePubOVX and Sham animals were killed by fast decapitation. The right hippocampi were dissected using Paxinos coordinates as described previously (21). Unpublished data from a previous microarray study in our lab indicate no significant hemisphere-specific differences in the DEGs between the two strains. Further, we found no hemisphere-specific differences of these genes in the Allen Brain Atlas. Total RNA from individual right hippocampi was extracted with the RNeasy Lipid Tissue Mini Kit (Qiagen, Santa Clarita, CA, USA) according to the manufacturer’s protocol, including treatment with DNase.

### Construction of RNA-Sequencing (RNA-Seq) Libraries

RNA-sequencing was performed from hippocampal RNA (*N* = 4/strain/treatment group). RNA quality was evaluated with an Agilent 2100 Bioanalyzer (Agilent Technologies). Libraries were created from high quality RNA (RNA integrity number of 7 or greater) using the SENSE mRNA-Seq Library Prep Kit from Lexogen (Lexogen, Vienna, Austria) according to the manufacturer’s protocol. This protocol includes an integrated poly(A) mRNA selection process, and the constructed library maintains the strand specificity. One single RNA-Seq library was constructed for each RNA sample. Library production is initiated by the random hybridization of Starter/Stopper heterodimers to the RNA template, followed by first and second strand cDNA synthesis. The cDNA was subject to end repair, adenylation of 3′ ends and adapter ligation. The adapters contained a unique “barcode” for each sample, which allows for multiplexing. The cDNA library quality and size distribution were checked using an Agilent Bioanalyzer and DNA 1000 chip. Library fragment sizes were between 200 and 500 bp, with a peak at B260 bp. All libraries were quantified with a Qubit 2.0 Fluorometer (Life Technologies) and stored at −20°C.

### Sequencing and Analysis

RNA-sequencing libraries were sequenced using Life Technologies 5500XL Wildfire Genetic Analyzer. Samples were equally distributed between and multiplexed in three lanes and sequenced with 50 bp single end reads. Raw sequencing data have been deposited in the NCBI Short Read Archive^1^ (SRA accession number SAMN07569379). The sequencing reads were first converted from the *xsq* format to *csfasta* format. Read quality was stored in *qual* files. The SHRiMP (v2.2.3) software was used to align the sequences to the rat reference genome (rn5) using the default parameters. Sequence reads spanning the exonal junctions were recovered (based on the CIGAR code) using TopHat (v1.4.1) (41) and the RefSeq database as a reference. The genomic and exonal junction reads were then combined into a single sorted *bam* file for each sample, with reads from the different sequencing lanes remaining separated. HTSeq was then used to count the number of reads, mapped to each gene using the RefSeq database as the reference. DESeq2, of the Bioconductor suite, was used for differential expression analysis. Read counts were normalized according to the DESeq2 procedure and DEGs were identified between groups while controlling for any variations associated with the sequencing lane. In any comparison, at least one group had to have an average of transcript reads >10, to reduce false positives due to very low read counts. Highly significant DEGs are defined as those with a *q* value <0.05 and fold change (FC) below 0.7 or above 1.3.

The average total reads mapped per sample group were WLI PrePubOVX: 48,285,977 ± 3,179,918, WLI Sham: 47,622,998 ± 5,372,814, WMI PrePubOVX: 54,551,134 ± 5,305,802, and WMI Sham: 51,868,942 ± 1,858,496. The percent unique reads per sample group were as follows: WLI PrePubOVX: 50.84 ± 0.86%, WLI Sham: 49.99 ± 0.71%, WMI PrePubOVX: 49.01 ± 0.74%, and WMI Sham: 49.45 ± 0.745%.

### Gene Ontology and Pathway Analysis

Pathway and global functional analyses were performed using Ingenuity Pathway Analysis 6.0^2^ (IPA). A data set containing gene identifiers and corresponding expression values was uploaded into the application, and each gene identifier was mapped to its corresponding gene object in the ingenuity pathways knowledge base. The functional and canonical pathway analysis identified interacting networks associated with DEGs between groups. The significance of the association between the data set and the network was measured by the *p*-value of the Fisher’s exact test. Because of the sample size per group and the multiple comparisons needed for testing the hypotheses, the cutoff *p*-value was set at *p* < 0.01 to balance type 1 and type 2 errors in the initial analyses for the Ingenuity Pathway Analysis. We focused on the most significant network from each comparison.

### Real-time Reverse Transcription-Polymerase Chain Reaction (RT-qPCR)

RT-qPCR was used to corroborate the RNA-Seq findings using randomly selected transcripts. RNA samples from the right hippocampus of EA (PN 32–34) WMIs and WLIs, obtained in our previous study (21), were also used in some of the qPCR analyses to compare hippocampal levels among EA, adult PrePubOVX and Sham females. cDNA was synthesized and RT-qPCR was performed on six to eight samples per strain/group as described previously (29). Briefly, the ABI 7900HT real-time cycler was used to amplify 5 ng cDNA using SYBR green reaction mix (ABI, Carlsbad, CA, USA). Primers were designed to amplify 80–150 bp products using the default settings of ABI’s Primer Express software (version 3.0, PE Applied Biosystems). Primer pairs are listed in Table S1 in Supplementary Material. Reactions were performed in triplicate and reached threshold amplification within 35 PCR cycles. Transcript levels were determined relative to GAPDH using the 2^−(ΔΔCt)^ method.

### Statistical Analysis

Data points were removed only for technical reasons, such as when the TSE program could not track the animals’ movement in the OFT, or when the quantitative RT-qPCR results were uninterpretable. All data were analyzed using GraphPad Prism v 7 (GraphPad Software, La Jolla, CA, USA). Two-way ANOVA determined significant differences between the strains (WMI vs. WLI) and conditions (Sham vs. PrePubOVX) for all behavioral measures and for most RT-qPCR analyses. Data were tested for normality and homogeneity using the D’Agostino and Pearson normality test and the homogeneity of variance test. Based on the recommendation of GraphPad, data were transformed when these tests showed highly significant deviation from Gaussian distribution. Bonferroni-corrected significance or hypothesis testing by Student’s *t*-test was reported in the figures. Significance was considered at *p* < 0.05. Data are presented as mean ± SEM.

A trend of significance was identified at *p* < 0.1. In addition, we also detected *p* < 0.05 significance at *post hoc* comparisons, even when the ANOVA was not significant. Our decision to describe these results were based on an increasing number of discussions arguing that *p*-values are not as reliable as it is thought previously (42) and that while a three-group comparison ANOVA may not result in significance, two groups of the three can differ from each other at the *p* < 0.05 level (43).

Pearson correlation of FCs between the RNA-Seq and the qRT-PCR results was calculated for specific transcripts as described.

## Results

### Body Weight and Affective Behaviors

**Figure 1:**
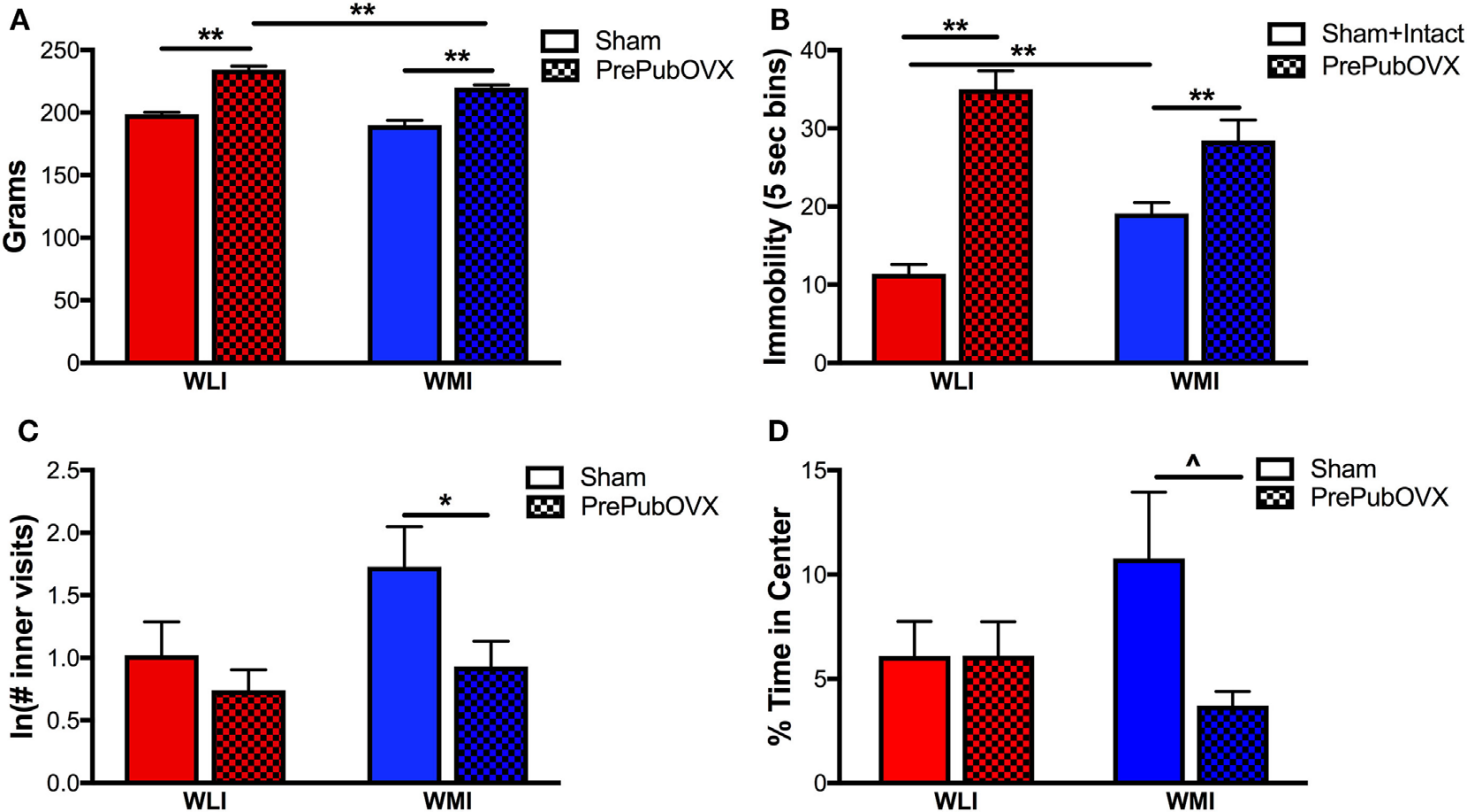
Removal of ovarian hormones before puberty alters body weight, depression- and anxiety-like behaviors. **(A)** Body weights as measured in grams in Sham and prepubertally ovariectomized adult female Wistar Kyoto (WKY) less immobile (WLIs) and WKY more immobiles (WMIs). **(B)** Immobility behavior in the forced swim test measured every 5 s (5 s bins). Immobility of intact animals did not differ from those of shams’ and therefore, data were combined. **(C)** Natural logarithm of the number of visits to the inner area of the open field arena and **(D)** percent time spent in the inner area of the open field arena. Lines marked by **p < 0.01 and *p < 0.05 reflect Bonferroni-corrected *post hoc* analyses. ^^^*p* < 0.05 by Student’s *t*-test, as hypothesis testing between two groups, when ANOVA is significant or a trend (*p* < 0.1). Data are presented as mean ± SEM.

In general, adult WLI females were heavier than WMIs [Strain: *F*(1,50) = 16.57, *p* < 0.01, Figure 1A]. Body weights of the PrePubOVX adults were significantly greater compared with Sham females and WLI PrePubOVX females were heavier than the WMI PrePubOVX adults [strain: *F*(1,50) = 16.6, *p* < 0.01; condition: *F*(1,50) = 133.5, *p* < 0.01]. Specifically, both WMI and WLI Sham females weigh less (*p* < 0.01) than their PrePubOVX counterparts.

Depression-like behavior was assessed by time spent being immobile on the second day of the FST test (Figure 1B). Sham surgery had no effect on the FST immobility of adult randomly cycling females of either strain, therefore, Sham and intact data for FST were combined. As expected, Sham and intact WMIs showed greater immobility than WLIs (21, 29). In general, ovariectomy before puberty increased the immobility of adult animals [condition: *F*(1,86) = 79.93, *p* < 0.01]. Floating was significantly greater in the PrePubOVX animals of both the WMI and WLI strain (*p* < 0.01). However, the degree of this increase differed by strain [strain × condition: *F*(1,86) = 15.04, *p* < 0.01]. Specifically, PrePubOVX resulted in a significantly greater change from controls in WLI’s immobility compared with WMIs [23.63 ± 2.64 vs. 9.33 ± 2.98; *t*(27) = 3.55, *p* < 0.01].

Increased anxiety-like behavior was observed in the WMI PrePubOVX adults compared with WMI Shams in the open field test. As the inner visit measure showed deviation from Gaussian distribution, the ln transformation of the data was analyzed and shown on Figure 1C. WMI PrePubOVX females exhibited decreased number of inner visits [Figure 1C; condition: *F*(1,50) = 5.12, *p* < 0.05]. Anxiety-like behavior, as measured by % time spent in the center of the open field, tended to increase in the PrePubOVX adults compared with Shams [Figure 1D; condition: *F*(1,42) = 2.88, *p* = 0.09], this was driven by increased anxiety-like behavior of the PrePubOVX WMIs compared with WMI Shams, only [*t*(17) = 2.28, *p* < 0.05].

### Differential Gene Expression

To study transcriptional regulation in the PrePubOVX hippocampus of both strains and identify DEGs that may underlie enhanced despair- and/or anxiety-like behaviors, we next performed RNA-Seq analysis. Genes appearing in Tables 1–4 include a list of significant genes where one comparison resulted in a *q* value (false discovery rate) <0.05.

**Table 1:** Wistar Kyoto (WKY) less immobile (WLI) Sham vs. WKY more immobile (WMI) sham, differentially expressed genes at FDR < 0.05; Fold change (FC) > 1.3 or fold change < 0.7.

Gene symbol	Description	FC
*Frk*^3,b,c*^	Fyn-related Src family tyrosine kinase	0.317
*Cdhr5*	Cadherin-related family member 5	0.366
*Dpp6*^3,b,c*^	Dipeptidylpeptidase 6	0.371
*Dph5*	Diphthamide biosynthesis 5	0.486
*Cnppd1*	Cyclin Pas1/PHO80 domain containing 1	0.488
*Usp6nl*^3,b,c*^	USP6 N-terminal like	0.515
*Nphp3*	Nephronophthisis 3 (adolescent)	0.515
*RT1-A3*	RT1 class I, locus A3	0.519
*Mppe1*	Metallophosphoesterase 1	0.563
*Tspan7*^3,b,c*^	Tetraspanin 7 pseudogene	0.569
*Il9r*^2,a,d^	Interleukin 9 receptor	0.605
*Dpy19l3*^3,b,c*^	Dpy-19-like 3 (*C. elegans*)	0.620
*Pcnx*	Pecanex homolog (*Drosophila*)	0.630
*Slc22a7*^2,a,d^	Solute carrier family 22 (organic anion transporter), member 7	0.651
*Nme7*^2,a,d^	NME/NM23 family member 7	0.669
*Traf6*^3,b,c*^	TNF receptor-associated factor 6, E3 ubiquitin protein ligase	1.343
*Tcte1*	T-complex-associated testis expressed 1	1.349
*Ist1*	Increased sodium tolerance 1 homolog (yeast)	1.426
*Slc22a24*	Solute carrier family 22, member 24	1.491
*Cacna1e*	Calcium channel, voltage-dependent, R type, alpha 1E subunit	1.509
*Rsbn1l*	Round spermatid basic protein 1-like	1.520
*Zc3h6*	Zinc finger CCCH type containing 6	1.535
*Cd96*	CD96 molecule	1.538
*RGD1304694*^3,b,c*^	Similar to CG9646-PA	1.613
*Tnfrsf4*	Tumor necrosis factor receptor superfamily, member 4	1.633
*Sec63*	SEC63 homolog (*S. cerevisiae*)	1.642
*Papola*	Poly (A) polymerase alpha	1.719
*Qrich1*^2,b,d^	Glutamine-rich 1	1.761
*Slc35c2*^2,b,d^	Solute carrier family 35 (GDP-fucose transporter), member C2	1.802
*Map3k2*	Mitogen-activated protein kinase kinase kinase 2	1.806
*Celsr3*^2,b,d^	Cadherin, EGF LAG seven-pass G-type receptor 3	1.820
*Actbl2*	Actin, beta-like 2	1.944
*Casc5*^2,b,d^	Cancer susceptibility candidate 5	2.034
*Clec9a*^2,b,d^	C-type lectin domain family 9, member A	2.041
*Cabin1*^2,b,d^	Calcineurin binding protein 1	2.060

*^2^Overlap with WLI PrePubOVX vs. WLI Sham*.

*^3^Overlap with WMI PrePubOVX vs. WMI Sham*.

*^a^Change in expression parallels change in forced swim test (FST) behavior*.

*^b^Change in expression does not parallel change in FST behavior*.

^c^Change in expression parallels change in open field test (OFT) behavior; *Parallel or inverse.

*^d^Change in expression does not parallel change in OFT behavior*.

**Table 2:** Wistar Kyoto (WKY) less immobile (WLI) PrePubOVX vs. WLI Sham, differentially expressed genes at FDR < 0.05; Fold change (FC) > 1.3 or fold change < 0.7.

Gene symbol	Description	FC
*Clec9a*^1,b,d^	C-type lectin domain family 9, member A	0.490
*Slc35c2*^1,b,d^	Solute carrier family 35 (GDP-fucose transporter), member C2	0.502
*Casc5*^1,b,d^	Cancer susceptibility candidate 5	0.531
*Cabin1*^1,b,d^	Calcineurin binding protein 1	0.535
*Celsr3*^1,b,d^	Cadherin, EGF LAG seven-pass G-type receptor 3	0.577
*Qrich1*^1,b,d^	Glutamine-rich 1	0.585
*Golga4*	Golgin A4	0.598
*Dnajc10*	DnaJ (Hsp40) homolog, subfamily C, member 10	0.601
*Sdc3*	Syndecan 3	0.617
*Pprc1*	Peroxisome proliferator-activated receptor gamma, coactivator-related 1	0.663
*Ccdc175*	Coiled-coil domain containing 175	0.677
*Foxh1*	Forkhead box H1	1.300
*Rnf215*	Ring finger protein 215	1.303
*Stub1*	STIP1 homology and U-box containing protein 1, E3 ubiquitin protein ligase	1.308
*Kif20a*	Kinesin family member 20A	1.325
*mrpl24*	Mitochondrial ribosomal protein L24	1.331
*Ctsa*	Cathepsin A	1.341
*Anp32a*	Acidic (leucine-rich) nuclear phosphoprotein 32 family, member A	1.341
*Bace1*	Beta-site APP cleaving enzyme 1	1.365
*Dalrd3*	DALR anticodon binding domain containing 3	1.367
*Mars*	Methionyl-tRNA synthetase	1.369
*Dnajc14*	DnaJ (Hsp40) homolog, subfamily C, member 14	1.369
*Tp73*	Tumor protein p73	1.380
*Rai1*	Retinoic acid induced 1	1.388
*Ppip5k1*	Diphosphoinositol pentakisphosphate kinase 1	1.389
*Aip*	Aryl-hydrocarbon receptor-interacting protein	1.390
*Tmem88*	Transmembrane protein 88	1.427
*Angptl6*	Angiopoietin-like 6	1.428
*Capn12*	Calpain 12	1.433
*Thra*	Thyroid hormone receptor alpha	1.437
*Cers4*	Ceramide synthase 4	1.445
*Frem3*	FRAS1-related extracellular matrix 3	1.447
*B4galnt1*	Beta-1,4-*N*-acetyl-galactosaminyl transferase 1	1.447
*Ccdc24*	Coiled-coil domain containing 24	1.460
*H2afy2*	H2A histone family, member Y2	1.465
*Hsp90aa1*	Heat shock protein 90, alpha (cytosolic), class A member 1	1.472
*Klc3*	Kinesin light chain 3	1.477
*Tenm4*	Teneurin transmembrane protein 4	1.478
*Ptger1*	Prostaglandin E receptor 1 (subtype EP1)	1.486
*Tmed1*	Transmembrane emp24 protein transport domain containing 1	1.487
*Il9r*^1,a,d^	Interleukin 9 receptor	1.500
*Creb3*	cAMP responsive element binding protein 3	1.506
*Pdzd7*	PDZ domain containing 7	1.509
*Gatc*	Glutamyl-tRNA(Gln) amidotransferase, subunit C	1.512
*Glg1*	Golgi glycoprotein 1	1.517
*Cd3eap*	CD3e molecule, epsilon associated protein	1.522
*Dda1*	DET1 and DDB1 associated 1	1.522
*Esrra*	Estrogen-related receptor, alpha	1.524
*Zfhx2*	Zinc finger homeobox 2	1.526
*Gabra2*	Gamma-aminobutyric acid (GABA) A receptor, alpha 2	1.551
*Tmem88*	Transmembrane protein 88	1.571
*Smarcd3*	SWI/SNF related, matrix associated, actin dependent regulator of chromatin, subfamily d, member 3	1.579
*Ypel4*	Yippee-like 4 (*Drosophila*)	1.581
*Ttyh3*	Tweety family member 3	1.587
*Gpat2*	Glycerol-3-phosphate acyltransferase 2, mitochondrial	1.614
*Inpp5a*	Inositol polyphosphate-5-phosphatase A	1.623
*Samhd1*	SAM domain and HD domain, 1	1.624
*Nr3c1*	Nuclear receptor subfamily 3, group C, member 1	1.627
*Nme7*^1,a,d^	NME/NM23 family member 7	1.630
*Pmel*	Premelanosome protein	1.662
*Camlg*	Calcium modulating ligand	1.681
*Doc2g*	Double C2-like domains, gamma	1.687
*Morf4l1*	Mortality factor 4 like 1	1.748
*Cyp21a1*	Cytochrome P450, family 21, subfamily a, polypeptide 1	1.774
*RT1-T24-2*	RT1 class I, locus T24, gene 2	1.786
*Pth1r*	Parathyroid hormone 1 receptor	1.831
*Topors*	Topoisomerase I binding, arginine/serine-rich, E3 ubiquitin protein ligase	1.910
*Slc22a7*^1,a,d^	Solute carrier family 22 (organic anion transporter), member 7	1.940
*Pigh*^4^	Phosphatidylinositol glycan anchor biosynthesis, class H	1.947
*Myh7*	Myosin, heavy chain 7, cardiac muscle, beta	2.150
*Myh6*	Myosin, heavy chain 6, cardiac muscle, alpha	2.169
*Anks1b*^4^	Ankyrin repeat and sterile alpha motif domain containing 1B	2.207
*Nrk*^4^	Nik-related kinase	2.398

*^1^Overlap with WLI Sham vs. WKY more immobile (WMI) Sham*.

*^4^Overlap with WLI PrePubOVX vs. WMI PrePubOVX*.

*^a^Change in expression parallels change in forced swim test (FST) behavior*.

*^b^Change in expression does not parallel change in FST behavior*.

*^d^Change in expression does not parallel change in OFT behavior*.

**Table 3:** Wistar Kyoto (WKY) more immobile (WMI) PrePubOVX vs. WMI Sham, differentially expressed genes at FDR < 0.05; Fold change (FC) > 1.3 or fold change < 0.7.

Gene symbol	Description	FC
*Jmjd6*	Jumonji domain containing 6	0.285
*Frk*^1,b,c*^	Fyn-related Src family tyrosine kinase	0.379
*Dpp6*^1,b,c*^	Dipeptidylpeptidase 6	0.394
*Usp6nl*^1,b,c*^	USP6 N-terminal like	0.507
*Tspan7*^1,b,c*^	Tetraspanin 7 pseudogene	0.571
*Mettl5*	Methyltransferase like 5	0.615
*Dpy19l3*^1,b,c*^	Dpy-19-like 3 (*C. elegans*)	0.620
*Ccdc42*	Coiled-coil domain containing 42	0.645
*Uba2*	Ubiquitin-like modifier activating enzyme 2	0.653
*Proca1*	Protein interacting with cyclin A1	0.685
*Nemf*	Nuclear export mediator factor	0.695
Traf6^1,b,c*^	TNF receptor-associated factor 6, E3 ubiquitin protein ligase	1.376
*Phrf1*	PHD and ring finger domains 1	1.501
*Fam131b*	Family with sequence similarity 131, member B	1.521
*Tmem200c*	Transmembrane protein 200C	1.524
*Ano6*	Anoctamin 6	1.690
*RGD1304694*^1,b,c*^	Similar to CG9646-PA	1.691
*Zfp36l2*	Zinc finger protein 36, C3H type-like 2	1.704
*Marcks*^4^	Myristoylated alanine rich protein kinase C substrate	2.339
*Cep104*^4^	Centrosomal protein 104	2.432
*Slc9a3r2*^4^	Solute carrier family 9, subfamily A (NHE3, cation proton antiporter 3), member 3 regulator 2	2.834

*^1^Overlap with WKY less immobile (WLI) Sham vs. WMI Sham*.

*^4^Overlap with WLI PrePubOVX vs. WMI PrePubOVX*.

*^b^Change in expression does not parallel change in FST behavior*.

^c^Change in expression parallels change in open field test (OFT) behavior; ^*^Parallel or inverse.

**Table 4:** Wistar Kyoto (WKY) less immobile (WLI) PrePubOVX vs. WKY more immobile (WMI) PrePubOVX differentially expressed genes at FDR < 0.05; Fold change (FC) > 1.3 or fold change < 0.7.

Gene symbol	Description	FC
*Zdhhc18*	Zinc finger, DHHC-type containing 18	0.219
*Nrk*	Nik-related kinase	0.499
*Anks1b*^2^	Ankyrin repeat and sterile alpha motif domain containing 1B	0.517
*Fanca*	Fanconi anemia, complementation group A	0.598
*Pigh*^2^	Phosphatidylinositol glycan anchor biosynthesis, class H	0.601
*Marcks*^3^	Myristoylated alanine rich protein kinase C substrate	2.148
*Rtkn2*	Rhotekin 2	2.175
*Prkci*	Protein kinase C, iota	2.192
*Cep104*^3^	Centrosomal protein 104	2.263
*Nacc1*	Nucleus accumbens associated 1, BEN and BTB (POZ) domain containing	2.315
*Slc9a3r2*^3^	Solute carrier family 9, subfamily A (NHE3, cation proton antiporter 3), member 3 regulator 2	2.804

*^2^Overlap with WLI PrePubOVX vs. WLI Sham*.

*^3^Overlap with WMI PrePubOVX vs. WMI Sham*.

Of the 17,905 transcripts that were aligned to RefSeq IDs, 35 transcripts were significantly different between WLI Sham vs. WMI Sham (Table 1), with an fold change (FC) > 1.3 or fold change < 0.7. Seventy-three DEGs were significant between WLI PrePubOVX and WLI Sham (Table 2), of which 61 were unique to this comparison. Of the 21 DEGs that were significant in the WMI PrePubOVX vs. WMI Sham comparison (Table 3), 11 were unique to this comparison. Finally, the expression of only five transcripts differed between WLI PrePubOVX vs. WMI PrePubOVX, with no overlap with the other comparisons (Table 4). Figure S2 in Supplementary Material displays the FC and *p*-value relationship of transcripts in the WLI PrePubOVX and WMI PrePubOVX comparison.

Quantitative RT-PCR analyses of selected DEGs were carried out. The selection was based on the expression pattern that paralleled, linearly or inversely, either the FST or the OFT behavioral changes. Specifically, transcript levels were measured from hippocampal RNA for those genes that showed higher (or lower) levels in the WMI Sham compared with WLI Sham hippocampus simultaneously with higher (or lower) levels in the WLI PrePubOVX compared with WLI Sham; an FST profile (Tables 1 and 2). These are *Il9r, Nme7*, and *Slc22a7* (Figure S3A in Supplementary Material). Of these, expression of *Nme7* confirmed all the criteria by qPCR, as well as the increased expression of *Slc22a7* by PrePubOVX in the WLI strain. There were no transcripts that fulfilled both the higher (or lower) levels in the WMI Sham vs. WLI Sham and the higher (or lower) WMI PrePubOVX vs. WMI Sham comparisons.

For matching the OFT behavioral profile, no significant difference is needed between WLI and WMI Shams or WLI PrePubOVX and WLI Sham in their hippocampal transcript levels, but a significant difference in either direction between WMI PrePubOVX and WMI Sham (Tables 1–3.) These genes are *Traf6, RGD1304694, Frk, Dpp6, Usp6nl, Tspan7*, and *Dpy19l3* (Figure S3B in Supplementary Material), of which *Frk, Dpp6*, and *Dpy19l3* expression were confirmed. Since WLI PrePubOVX showed non-significantly greater immobility behavior in the FST compared with WMI PrePubOVX, we randomly selected some genes for qPCR measurements for the WLI PrePubOVX vs. WMI PrePubOVX comparison (Table 4). All three of these genes, *Pigh, Rtkn2*, and *Cep104* showed significant differences in their hippocampal expression between these groups (Figure S3C in Supplementary Material).

The correlation between the RNA-Seq FC (Tables 1–4) and their corresponding RT-qPCR FC was calculated for the genes shown in Figure S3 in Supplementary Material (*r* = 0.90, *p* < 0.01) (Figure S4 in Supplementary Material).

### Network Analyses Using IPA

The IPA canonical pathway analysis between the two strains under Sham conditions revealed significant alternations in pathways related to cellular movement (*Daxx, Frk, Dlg1, Map3k2, Mapk13*, and more) and immune cell trafficking (*Traf6, Tnfrsf4, HLA-A, Il9r, Ifngr1*, and more). The top network (Fisher’s *t*-test *p* = 10^−39^) is shown in Figure S5 in Supplementary Material. Interconnectedness by different kinases, the extracellular signal-regulated kinases (Erk1/2), c-Jun N-terminal kinase, P38 mitogen-activated protein kinase (P38 Mapk), phosphatidylinositide 3-kinase as well as interferon alpha and signal transducer and activator of transcription 5a and b (Stat5a and b) is also demonstrated.

Differentially expressed genes between WLI PrePubOVX and WLI Sham form the network (Fisher’s *t*-test *p* = 10^−40^) displayed in Figure S6 in Supplementary Material. Major hubs in this network are the nuclear factor kappa-light-chain enhancer of activated B cells (Nfκb) and the vascular endothelial factor (Vegf). Activating the inflammatory signaling pathway, Nfκb is involved in cytokine-induced depressive behavior (44). VEGF is implicated in neuronal survival, neuroprotection, differentiation, and axonal growth, and its levels are reduced in the cerebrospinal fluid of severely depressed patients (45).

The most significant network (Fisher’s *t*-test *p* = 10^−39^) produced from WMI PrePubOVX and WMI Sham DEGs is shown in Figure S7 in Supplementary Material. Major hubs in this network also include Nfκb, Erk1, P38 MAPK, and Casp8 (caspase 8, apoptosis-related cysteine peptidase). Functions of several DEGs in this network are related to energy homeostasis (*Tshr, Slc2a3*, and *Lepr*), or to neurodegeneration (*Nox1, Herpud1*, or C*asp8*). In addition, although expression of thyroid hormone receptor alpha (*Thra*) was decreased in both strains after prepubertal ovariectomy (see Figure S6 in Supplementary Material also), *Tshr* expression was upregulated only in the WMI PrePubOVX hippocampus compared with Sham. This implies a unique regulatory connection between *Thra* and *Tshr* in the WMI PrePubOVX hippocampus.

**Figure 2:**
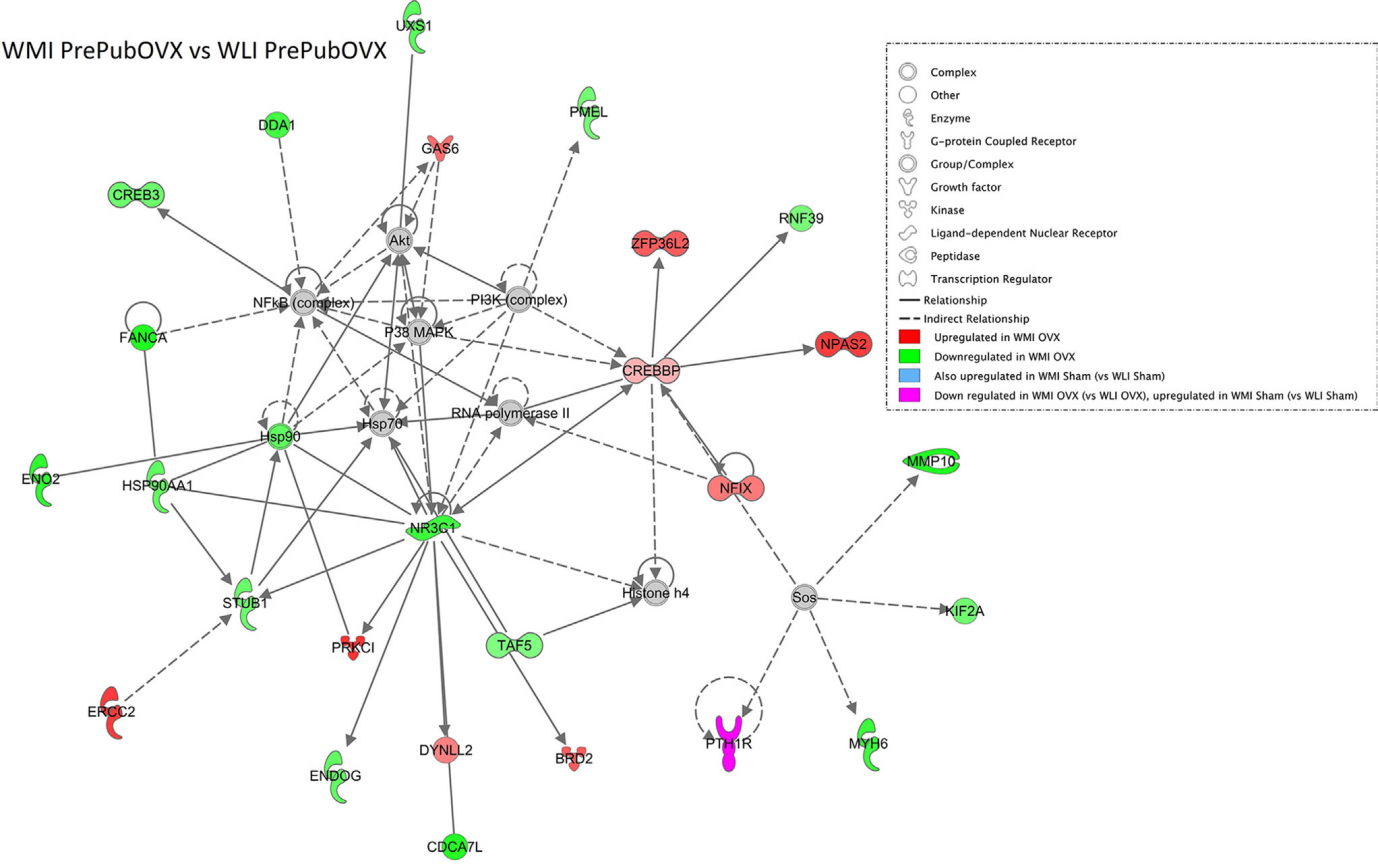
The most significant IPA Generated Network of Wistar Kyoto (WKY) more immobile (WMI) PrePubOVX vs. WKY less immobile (WLI) PrePubOVX differentially expressed genes (DEGs). DEGs with *p* < 0.01 were submitted to IPA with their corresponding fold changes using settings that allow for direct and indirect connections to other members of the network. Colored members are DEGs, while gray color indicates non-DEG connectors. The molecular/biological characteristics of the members are indicated in the legend.

Differentially expressed genes of the WMI PrePubOVX vs. WLI PrePubOVX comparison generate an IPA network (Fisher’s *t*-test *p* = 10^−41^) that shows a surprisingly large number of glucocorticoid signaling pathway regulators such as the glucocorticoid receptor (*Nr3c1*), *Crebbp, Hsp90, Hsp90aa1, Stub1, Endog, Dynll2*, and *Taf5* (Figure 2). It is also of interest that this network shows no overlap with the between strain, Sham comparison (WLI Sham vs. WMI Sham) network (Figure S5 in Supplementary Material).

**Figure 3:**
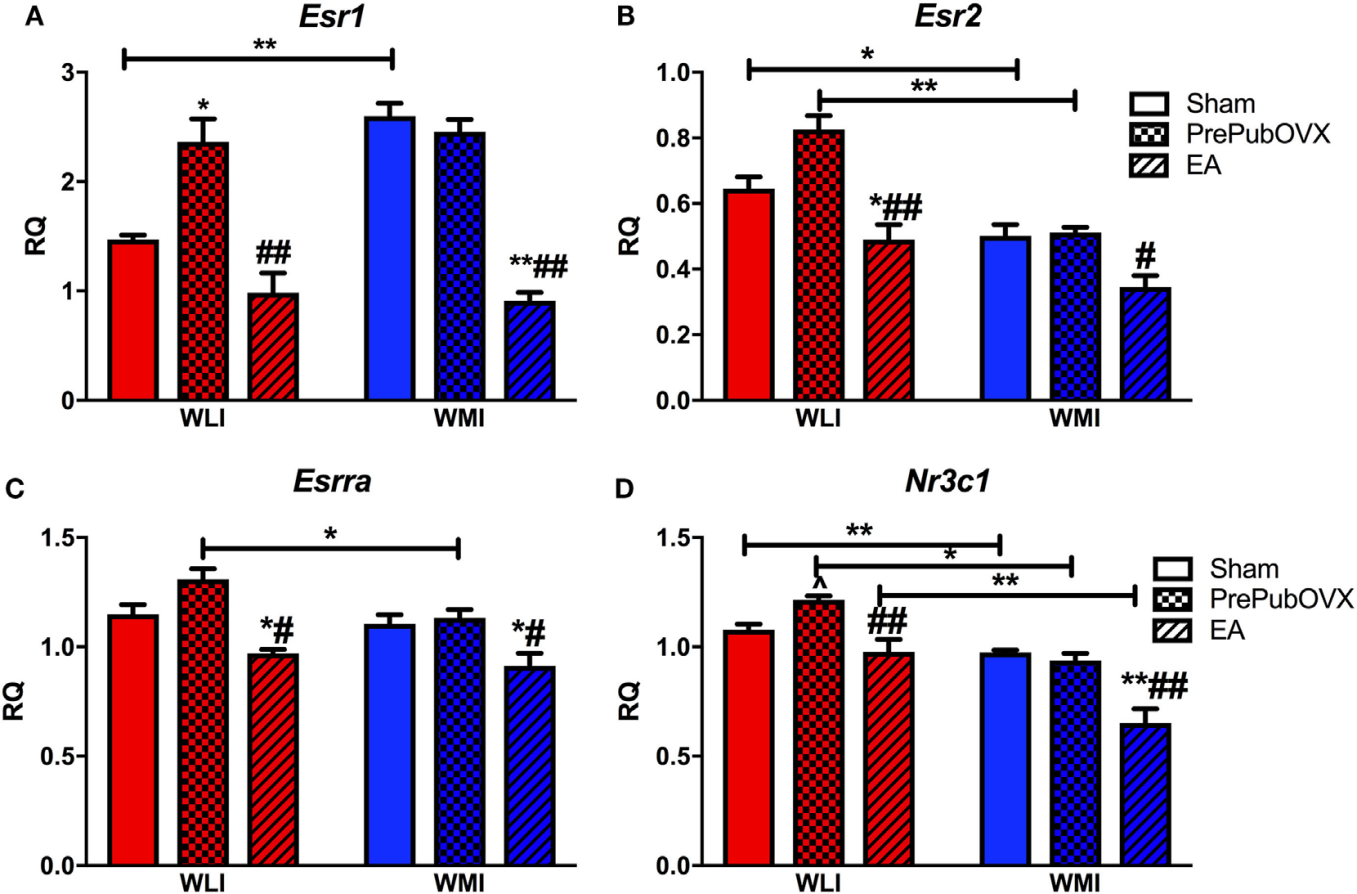
Lack of ovarian hormones before puberty [early adolescents (EA)] or by PrePubOVX affects hippocampal expression of estrogen and glucocorticoid receptors. Gene expression levels as measured by RT-qPCR. **(A)** Estrogen receptor alpha (*Esr1*). **(B)** Estrogen receptor beta (*Esr2*). **(C)** Estrogen-related receptor alpha (*Esrra*), and **(D)** glucocorticoid receptor (*Nr3c1*). Relative quantification (RQ) are Ct values normalized to GAPDH, and then to a universal calibrator calculated using the −ΔΔCt method. **p < 0.01 and *p < 0.05 are within strain group comparisons compared with Sham animals, ^##^*p* < 0.01 and ^#^*p* < 0.05 are within strain compared with PrePubOVX animals, lines marked by **p < 0.01 and *p < 0.05 are for strain comparisons, all by Bonferroni-corrected *post hoc* analyses. ^^^*p* < 0.05 by Student’s *t*-test, as hypothesis testing between two groups, when ANOVA is significant or a trend (*p* < 0.1). Data are presented as mean ± SEM.

As determined by RT-qPCR hippocampal estrogen receptor alpha (*Esr1*) transcript levels were significantly higher in WMIs than WLIs [strain, *F*(1,28) = 7.77, *p* < 0.01]. This difference originated from the transcript levels in Sham animals with no strain differences between the PrePubOVX adult females (Figure 3A). Specifically, while PrePubOVX increased *Esr1* expression in the WLI hippocampus compared with its Sham levels, it did not change it in the WMIs [condition, *F*(2,28) = 43.92, *p* < 0.01; strain × condition, *F*(2,28) = 6.69, *p* < 0.01]. EA *Esr1* levels were significantly lower in both strains of females compared with those of PrePubOVX. The major findings in the expression profile of estrogen receptor beta (*Esr2*) were the lower transcript levels of the Sham and PrePubOVX WMIs compared with their WLI counterparts [Figure 3B, strain, *F*(1,32) = 45.36, *p* < 0.01; condition, *F*(2,32) = 24.40, *p* < 0.01; strain × condition, *F*(2,32) = 3.65, *p* < 0.05]. This is the opposite of that of *Esr1* expression strain differences. Hippocampal expression of estrogen-related receptor—alpha (*Essra*) differed only by strain between adult PrePubOVX females, where the WLIs showed upregulation [Figure 3C, strain, *F*(1,33) = 7.47, *p* = 0.01; condition, *F*(2,33) = 22.69, *p* < 0.01]. Finally, glucocorticoid receptor (*Nr3c1*) transcript levels showed a condition-dependent expression profile very similar to that of *Esr2* in both strains (Figure 3D). Specifically, *Nr3c1* expression was lower in the WMI hippocampus compared with WLI [strain, *F*(1,31) = 53.67, *p* < 0.01] and was modified by ovarian hormone status [condition, *F*(2,31) = 24.42, *p* < 0.01]. This change differed between WLIs and WMIs, as *Nr3c1* transcript levels tended to increase by PrePubOVX only in WLIs, while EA transcript levels differed from the adult Shams’ only in the WMIs [strain × condition, *F*(2,31) = 4.49, *p* < 0.05].

## Discussion

This is the first study to assert behavioral and hippocampal molecular changes resulting from the interaction of genetic predisposition to depressive behavior with the lack of ovarian hormone regulated processes during adolescent development. Lack of ovarian hormones during and after puberty exaggerated both depression- and anxiety-like behaviors of the WMI females with genetic predisposition to despair-like behavior. In the absence of this genetic predisposition, prepubertal ovariectomy resulted in a dramatic increase in depression-like behavior without effects on anxiety-like behavior in the WLI control strain. Hippocampal transcriptomes of the two strains of animals that were Sham-ovariectomized did not differ dramatically, as expected, due to the genetic relatedness of these two strains. However, the two strains responded differently to prepubertal ovariectomy in their hippocampal transcriptomic changes. Furthermore, transcript level differences in estrogen and glucocorticoid receptors in the hippocampus of PrePubOVX vs. Sham females suggest that the lack of ovarian hormones affect the WLI females more profoundly, in parallel with the dramatic increase in their depression-like behavior.

In preclinical studies, there is evidence for antidepressive effects of E2. Removal of the E2 primary source by ovariectomy increases depressive behavior in the FST (14, 46–51), and E2 replacement reverses this effect. Thus, the rising levels of E2 at puberty should attenuate depression-like behavior, but instead, it is when female prevalence of depression becomes evident in humans and depression-like behavior is increased in the genetic model of depression, the WMI (21). Thus, the actions of E2, or ovarian hormones during puberty may differ from that in adulthood. In this study, the exaggerated depression-like behavior of the PrePubOVX females could be the result of the continuing absence of ovarian hormones during both puberty and in adulthood. Whether the effect of OVX during only the pubertal period would be the same will have to be determined in the future.

In women, anxiety is frequently comorbid with depression (52, 53), and this is reflected in most animal models of depression as they exhibit both behaviors. The WMIs and WLIs were bidirectionally selected based on depression-like behavior from the parental WKY strain, which shows both depression- and anxiety-like behaviors (22–24). Since we selected for depression-like behavior, anxiety-like behavior likely segregated independently as illustrated by the male WLIs showing higher anxiety-like behavior than WMIs, which is not modified by developmental age (21). While EA WMI females showed less anxiety-like behavior than WLI females similarly to males, adult female WMIs were as anxious as WLIs (21). Prepubertally ovariectomized WMIs showed greater anxiety-like behavior in the OFT than their Sham controls, while in contrast to depression-like behavior, removal of ovarian hormones prepubertally had no effect on the anxiety-like behavior of the WLIs. Other studies also report no effect of prepubertal ovariectomy on OFT behavior in a routinely used rat strain (13). By contrast, adult age ovariectomy increases anxiety-like behavior in rodents (54–56), and hormone replacement reduces these behaviors in ovariectomized female rats (54, 56–62). Whether the anxiolytic effects of ovarian hormones are more profound in adulthood than in puberty is another question this study cannot answer. Nevertheless, ovarian hormone-dependent processes seem to specifically affect the WMI strain.

Estrogen-dependent synaptogenesis, which could affect behavior, is known to depend on estrogen receptors, *Esr1* and *Esr2* or ERα and ERβ (63, 64). ERα is present in nuclear and extranuclear sites and inhibitory neurons (63). Extranuclear ERβ is present in principal cells and in a few inhibitory cells (64). Since the hippocampal expression profiles of *Er1* and *Er2* were the complete opposite between WLI and WMI Sham females, one would assume that their actions would also differ. It has been suggested that ERβ (Esr2) mediates the anxiolytic properties of estrogen (65), although expression of the hippocampal *Esr2* of WMIs did not parallel their behavior in the OFT. Interestingly, no change was found in hippocampal transcript levels of estrogen or glucocorticoid receptors in the WMI females in response to prepubertal ovariectomy. Whether that is due to a so far unresolved cause of resistance to estrogen or to glucocorticoids in this strain is not known, but definitely deserve further experimentation.

The RNA-Seq results provided insights into the transcriptomic differences in the hippocampus resulting from the strain differences and/or caused by prepubertal ovariectomy in both strains. One important finding is the small number of DEGs between the strains, which underlie the genetic closeness of these two strains generated from the semi-inbred WKY strain (39). Further, there was no overlap between the WLIs and WMIs DEGs in response to prepubertal ovariectomy. This suggests that the molecular processes resulting in the increased depression-like behavior after the removal of ovarian hormones prepubertally are different in the two strains. In addition, DEGs between the WMI PrePubOVX and WLI PrePubOVX do not overlap with the DEGs between Shams, intimating that even the ovarian hormone-independent components of the neurodevelopmental processes are different between these strains.

The evaluation of hippocampal gene expression changes that parallel the behavioral changes in the two strains after prepubertal ovariectomy revealed some DEGs with potential relevance to human behavior. For example, hippocampal expression of the solute carrier family 22 (organic anion transporter), member 7 (*Slc22A7*) was dramatically higher in the WLI PrePubOVX females compared their Sham counterparts, and sequence variation of this gene was suggested to contribute to autism spectrum disorder, ALS, or schizophrenia (66). Interestingly, *Dpp6* (dipeptidyl peptidase-like 6), with expression parallel to the increased anxiety of PrePubOVX WMI females is also a candidate gene for ALS (67). Sequence variation in the *Dpy19l3* (Dpy-19-like C-mannosyltransferase 3) gene has been suggested to contribute to bipolar disorder (68). While these findings are not directly relevant to the current goals of the study, they indicate that these seemingly disparate biochemical entities can have behavioral relevance.

A less surprising finding is that the highly connected genes in the WMI PrePubOVX vs. WLI PrePubOVX network are members of the glucocorticoid signaling pathway. These include the glucocorticoid receptor (*Nr3c1*) and the essential components of glucocorticoid receptor function, *Hsp90* and *Hsp90aa1*, with decreased expression in the WMI Sham and PrePubOVX hippocampus compared with those of WLIs. *Crebbp*, which is a coactivator of the glucocorticoid receptor, can also function conditionally as a negative regulator of its activities. It is upregulated to a similar degree in both PrePubOVX and Sham WMIs, suggesting that *Crebbp* expression is related to the genetic differences between the strains and is not affected by ovarian hormones. Decreased functioning of glucocorticoid receptors has been heavily implicated in depression etiology (38), and because downregulation of *Nr3c1* occurs in the WMI genetic model regardless of ovarian status, this may be not be the responsible mechanism for the PrePubOVX-induced increase in depression-like behavior in the WMIs. Interestingly, this pattern is the same for the hippocampal expression of the estrogen receptors, none of which show change by PrePubOVX in WMIs. By contrast, expression of all of these receptors increases by PrePubOVX in the WLIs. The dramatic increase in depression-like behavior in the WLI females when ovarian hormones are absent seems to parallel the changes in glucocorticoid and estrogen receptor expression, while the moderate change in both depression- and anxiety-like behaviors in the WMI females seems to be independent of them. Whether this major difference in the regulation of these nuclear receptors reflect a difference in sensitivity to hormones, glucocorticoids. or estrogen, cannot be determined from this study.

Pubertal status may play a role in behaviors associated with stress (69), such as depression and anxiety, since puberty is a period with heightened sensitivity to stressors (70). Adolescent animals display greater hormonal stress responses compared with adults following various stressors (71–73), and the adolescent brain seems to be more responsive to the stress-related hormones than the adult brain (74). In addition, brain areas relevant to affective behaviors continue to mature during adolescence (75). Whether a change in ovarian hormonal milieu, continuing brain development, increased stress responsiveness, or a combination of these factors is responsible for the unique vulnerability to the development of affective disorders during puberty is unknown. Future studies administering ovarian hormones to ovariectomized adult females of both strains can aid in discerning some of the mechanisms of these behavioral changes. In those studies, additional brain regions such as the bed nucleus of stria terminalis, amygdala, and the hypothalamus, known to be involved in depression and hormonal regulation of behavior, will likely be explored. The current findings indicate that lack of ovarian hormones during development can exaggerate depression-like behavior in adult females with or without a genetic contribution to depression. The distinct prepubertal ovariectomy-induced transcriptomic profiles in the two strains further confirm that genetic vulnerability and hormonal state-induced vulnerability might act *via* different pathways to generate similar behavioral profiles.

## Ethics Statement

This study was carried out in accordance with the recommendations by the Guide for the Care and Use of Laboratory Animals, 9th edition. The protocol was approved by the Institutional Animal Care and Use Committee of Northwestern University.

## Author Contributions

The study was designed by ER and NR. The experiments were carried out by NR, WL, and SC. The data were analyzed by NR, HC, MS, WL, LW, and ER. The manuscript was written and edited by NR, HC, MS, WL, and ER. All the authors have approved the final version of the manuscript.

## Conflict of Interest Statement

The authors declare that the research was conducted in the absence of any commercial or financial relationships that could be construed as a potential conflict of interest.

## Footnotes

^1^http://www.ncbi.nlm.nih.gov/sra/.

^2^http://www.ingenuity.com.

## Supplementary Material

Figure S1 Schematics of the experimental design. Timings of prepubertal ovariectomy, behavioral testing, and hippocampal tissue collection are indicated. Puberty was marked to begin at postnatal day 34, by which time 20% of females demonstrate the criteria of puberty as described previously (Holehan AM, Merry BJ, The control of puberty in the dietary restricted female rat. *Mech Ageing Dev* (1985) 32:179–91.).

Figure S2 Differential RNA expression between Wistar Kyoto (WKY) less immobile (WLI) PrePubOVX and WKY more immobile (WMI) PrePubOVX hippocampus. Volcano plots showing −log (*p*-value) vs. log 2 fold change (FC) in RNA abundance in transcripts between the strains after PrePubOVX. Red circles denote significant differences (FDR corrected *p* < 0.05). FC cutoff is set at fold change (FC) > 1.3 or fold change < 0.7.

Figure S3 Quantitative RT-PCR analyses of differentially expressed genes (DEGs) selected to parallel behaviors in the forced swim test (FST) or open field test (OFT) **(A)** Wistar Kyoto (WKY) less immobile (WLI) Sham vs. WKY More immobile (WMI) Sham and WLI PrePubOVX vs. WLI Sham comparison, proposed to parallel WLI behavior in the FST: *Il9r*; *Nme7*; and *Slc22a7*. **(B)** WMI PrePubOVX vs. WMI Sham comparison, proposed to mirror their behavior in the OFT: *Traf6, RGD1304604, Frk, Dpp6, Usp6nl, Tspan7*, and *Dpy19l3*. **(C)** WLI PrePubOVX vs. WMI PrePubOVX comparison, with non-significant behavioral differences in the FST: *Pigh, Rtkn2*, and *Cep104*. Relative quantification (RQ) calculated using the 2^−ΔΔCt^ method, after normalizing to GAPDH and a general hippocampal calibrator sample. ***p* < 0.01 and **p* < 0.05 by Bonferroni-corrected *post hoc* analyses following a two-way ANOVA; ^^^^*p* < 0.01 hypothesis testing by Student’s *t*-test when ANOVA is significant or a trend (*p* < 0.1). Data are presented as mean ± SEM.

Figure S4 Significant Spearman correlation between RNA-sequencing (RNA-Seq) and RT-qPCR. Fold change determined by quantitative RT-PCR or RNA-Seq of genes shown on Figure S3 in Supplementary Material.

Figure S5 The most significant IPA Generated Network of Wistar Kyoto (WKY) more immobile (WMI) Sham vs. WKY less immobile (WLI) Sham differentially expressed genes (DEGs). DEGs with *p* < 0.01 were submitted to IPA with their corresponding fold changes using settings that allow for direct and indirect connections to other members of the network. Colored members are DEGs, while grey color indicates non-DEG connectors. The molecular/biological characteristics of the members are indicated in the legend.

Figure S6 The most significant IPA Generated Network Wistar Kyoto (WKY) less immobile (WLI) Sham vs. WLI PrePubOVX differentially expressed genes (DEGs). DEGs with *p* < 0.01 were submitted to IPA with their corresponding fold changes using settings that allow for direct and indirect connections to other members of the network. Colored members are DEGs, while grey color indicates non-DEG connectors. The molecular/biological characteristics of the members are indicated in the legend.

Figure S7 The most significant IPA Generated Network Wistar Kyoto (WKY) more immobile (WMI) Sham vs. WMI PrePubOVX differentially expressed genes (DEGs). DEGs with *p* < 0.01 were submitted to IPA with their corresponding fold changes using settings that allow for direct and indirect connections to other members of the network. Colored members are DEGs, while grey color indicates non-DEG connectors. The molecular/biological characteristics of the members are indicated in the legend.

## References

[1] Merikangas KR He JP Burstein M Swanson SA Avenevoli S Cui L Lifetime prevalence of mental disorders in U.S. adolescents: results from the National Comorbidity Survey Replication – Adolescent Supplement (NCS-A). J Am Acad Child Adolesc Psychiatry (2010) 49(10):980–9. 10.1016/j.jaac.2010.05.017 20855043 PMC2946114

[2] Breslau N Schultz L Peterson E. Sex differences in depression: a role for preexisting anxiety. Psychiatry Res (1995) 58(1):1–12. 10.1016/0165-1781(95)02765-O 8539307

[3] Patchev VK Hayashi S Orikasa C Almeida OF. Implications of estrogen-dependent brain organization for gender differences in hypothalamo-pituitary-adrenal regulation. FASEB J (1995) 9(5):419–23. 7896013 10.1096/fasebj.9.5.7896013

[4] Weiser MJ Handa RJ. Estrogen impairs glucocorticoid dependent negative feedback on the hypothalamic-pituitary-adrenal axis via estrogen receptor alpha within the hypothalamus. Neuroscience (2009) 159(2):883–95. 10.1016/j.neuroscience.2008.12.058 19166915 PMC5837863

[5] Deecher D Andree TH Sloan D Schechter LE. From menarche to menopause: exploring the underlying biology of depression in women experiencing hormonal changes. Psychoneuroendocrinology (2008) 33(1):3–17. 10.1016/j.psyneuen.2007.10.006 18063486

[6] Wharton W Gleason CE Olson SR Carlsson CM Asthana S. Neurobiological underpinnings of the estrogen – mood relationship. Curr Psychiatry Rev (2012) 8(3):247–56. 10.2174/157340012800792957 23990808 PMC3753111

[7] Walf AA Frye CA. A review and update of mechanisms of estrogen in the hippocampus and amygdala for anxiety and depression behavior. Neuropsychopharmacology (2006) 31(6):1097–111. 10.1038/sj.npp.1301067 16554740 PMC3624621

[8] Walf AA Frye CA. Rapid and estrogen receptor beta mediated actions in the hippocampus mediate some functional effects of estrogen. Steroids (2008) 73(9–10):997–1007. 10.1016/j.steroids.2008.01.025 18342348 PMC2459332

[9] Walf AA Frye CA. Estradiol reduces anxiety- and depression-like behavior of aged female mice. Physiol Behav (2010) 99(2):169–74. 10.1016/j.physbeh.2009.09.017 19804793 PMC3618443

[10] Imhof JT Coelho ZM Schmitt ML Morato GS Carobrez AP. Influence of gender and age on performance of rats in the elevated plus maze apparatus. Behav Brain Res (1993) 56(2):177–80. 10.1016/0166-4328(93)90036-P 8240712

[11] Slawecki CJ. Comparison of anxiety-like behavior in adolescent and adult Sprague-Dawley rats. Behav Neurosci (2005) 119(6):1477–83. 10.1037/0735-7044.119.6.1477 16420152

[12] Lynn DA Brown GR. The ontogeny of anxiety-like behavior in rats from adolescence to adulthood. Dev Psychobiol (2010) 52(8):731–9. 10.1002/dev.20468 21117243 PMC3061011

[13] Zimmerberg B Farley MJ. Sex differences in anxiety behavior in rats: role of gonadal hormones. Physiol Behav (1993) 54(6):1119–24. 10.1016/0031-9384(93)90335-D 8295951

[14] Bernardi M Vergoni AV Sandrini M Tagliavini S Bertolini A. Influence of ovariectomy, estradiol and progesterone on the behavior of mice in an experimental model of depression. Physiol Behav (1989) 45(5):1067–8. 10.1016/0031-9384(89)90238-2 2780868

[15] Jenkins JA Williams P Kramer GL Davis LL Petty F. The influence of gender and the estrous cycle on learned helplessness in the rat. Biol Psychol (2001) 58(2):147–58. 10.1016/S0301-0511(01)00111-9 11600242

[16] Frye CA Walf AA. Changes in progesterone metabolites in the hippocampus can modulate open field and forced swim test behavior of proestrous rats. Horm Behav (2002) 41(3):306–15. 10.1006/hbeh.2002.1763 11971664

[17] CONVERGE Consortium. Sparse whole-genome sequencing identifies two loci for major depressive disorder. Nature (2015) 523(7562):588–91. 10.1038/nature14659 26176920 PMC4522619

[18] Kendler KS Prescott CA. A population-based twin study of lifetime major depression in men and women. Arch Gen Psychiatry (1999) 56(1):39–44. 10.1001/archpsyc.56.1.39 9892254

[19] Solberg LC Baum AE Ahmadiyeh N Shimomura K Li R Turek FW Sex- and lineage-specific inheritance of depression-like behavior in the rat. Mamm Genome (2004) 15(8):648–62. 10.1007/s00335-004-2326-z 15457344 PMC3764448

[20] Flint J Kendler KS. The genetics of major depression. Neuron (2014) 81(3):484–503. 10.1016/j.neuron.2014.01.027 24507187 PMC3919201

[21] Mehta NS Wang L Redei EE. Sex differences in depressive, anxious behaviors and hippocampal transcript levels in a genetic rat model. Genes Brain Behav (2013) 12(7):695–704. 10.1111/gbb.12063 23876038

[22] Pare WP Redei E Sex differences and stress response of WKY rats. Physiol Behav (1993) 54(6):1179–85. 10.1016/0031-9384(93)90345-G 8295961

[23] Pare WP Redei E Depressive behavior and stress ulcer in Wistar Kyoto rats. J Physiol Paris (1993) 87(4):229–38. 10.1016/0928-4257(93)90010-Q 8136789

[24] Pare WP. Open field, learned helplessness, conditioned defensive burying, and forced-swim tests in WKY rats. Physiol Behav (1994) 55(3):433–9. 10.1016/0031-9384(94)90097-3 8190758

[25] Solberg LC Olson SL Turek FW Redei E. Altered hormone levels and circadian rhythm of activity in the WKY rat, a putative animal model of depression. Am J Physiol Regul Integr Comp Physiol (2001) 281(3):R786–94. 10.1152/ajpregu.2001.281.3.R786 11506993

[26] Baum AE Solberg LC Churchill GA Ahmadiyeh N Takahashi JS Redei EE. Test- and behavior-specific genetic factors affect WKY hypoactivity in tests of emotionality. Behav Brain Res (2006) 169(2):220–30. 10.1016/j.bbr.2006.01.007 16490266 PMC3762875

[27] Malkesman O Braw Y Zagoory-Sharon O Golan O Lavi-Avnon Y Schroeder M Reward and anxiety in genetic animal models of childhood depression. Behav Brain Res (2005) 164(1):1–10. 10.1016/j.bbr.2005.04.023 16055204

[28] Malkesman O Braw Y Maayan R Weizman A Overstreet DH Shabat-Simon M Two different putative genetic animal models of childhood depression. Biol Psychiatry (2006) 59(1):17–23. 10.1016/j.biopsych.2005.05.039 16095569

[29] Andrus BM Blizinsky K Vedell PT Dennis K Shukla PK Schaffer DJ Gene expression patterns in the hippocampus and amygdala of endogenous depression and chronic stress models. Mol Psychiatry (2012) 17(1):49–61. 10.1038/mp.2010.119 21079605 PMC3117129

[30] Mehta-Raghavan NS Wert SL Morley C Graf EN Redei EE. Nature and nurture: environmental influences on a genetic rat model of depression. Transl Psychiatry (2016) 6:e770. 10.1038/tp.2016.28 27023176 PMC4872452

[31] Campbell S Macqueen G. The role of the hippocampus in the pathophysiology of major depression. J Psychiatry Neurosci (2004) 29(6):417–26. 15644983 PMC524959

[32] Arnone D McKie S Elliott R Juhasz G Thomas EJ Downey D State-dependent changes in hippocampal grey matter in depression. Mol Psychiatry (2013) 18(12):1265–72. 10.1038/mp.2012.150 23128153

[33] Curlik DM II Difeo G Shors TJ. Preparing for adulthood: thousands upon thousands of new cells are born in the hippocampus during puberty, and most survive with effortful learning. Front Neurosci (2014) 8:70. 10.3389/fnins.2014.00070 24795549 PMC4005956

[34] Bremner JD Narayan M Anderson ER Staib LH Miller HL Charney DS. Hippocampal volume reduction in major depression. Am J Psychiatry (2000) 157(1):115–8. 10.1176/ajp.157.1.115 10618023

[35] Greicius MD Flores BH Menon V Glover GH Solvason HB Kenna H Resting-state functional connectivity in major depression: abnormally increased contributions from subgenual cingulate cortex and thalamus. Biol Psychiatry (2007) 62(5):429–37. 10.1016/j.biopsych.2006.09.020 17210143 PMC2001244

[36] Williams KA Mehta NS Redei EE Wang L Procissi D. Aberrant resting-state functional connectivity in a genetic rat model of depression. Psychiatry Res (2014) 222(1–2):111–3. 10.1016/j.pscychresns.2014.02.001 24613017

[37] Gormley S Rouine J McIntosh A Kerskens C Harkin A. Glial fibrillary acidic protein (GFAP) immunoreactivity correlates with cortical perfusion parameters determined by bolus tracking arterial spin labelling (bt-ASL) magnetic resonance (MR) imaging in the Wistar Kyoto rat. Physiol Behav (2016) 160:66–79. 10.1016/j.physbeh.2016.04.007 27068181

[38] Pariante CM Miller AH. Glucocorticoid receptors in major depression: relevance to pathophysiology and treatment. Biol Psychiatry (2001) 49(5):391–404. 10.1016/S0006-3223(00)01088-X 11274650

[39] Will CC Aird F Redei EE. Selectively bred Wistar-Kyoto rats: an animal model of depression and hyper-responsiveness to antidepressants. Mol Psychiatry (2003) 8(11):925–32. 10.1038/sj.mp.4001345 14593430

[40] Castellano JM Bentsen AH Sanchez-Garrido MA Ruiz-Pino F Romero M Garcia-Galiano D Early metabolic programming of puberty onset: impact of changes in postnatal feeding and rearing conditions on the timing of puberty and development of the hypothalamic kisspeptin system. Endocrinology (2011) 152(9):3396–408. 10.1210/en.2010-1415 21712362

[41] Kim D Pertea G Trapnell C Pimentel H Kelley R Salzberg SL. TopHat2: accurate alignment of transcriptomes in the presence of insertions, deletions and gene fusions. Genome Biol (2013) 14(4):R36. 10.1186/gb-2013-14-4-r36 23618408 PMC4053844

[42] Nuzzo R Scientific method: statistical errors. Nature (2014) 506(7487):150–2. 10.1038/506150a 24522584

[43] Hsu J Multiple Comparisons: Theory and Methods. London: Chapman & Hall (1996).

[44] Nadjar A Bluthe RM May MJ Dantzer R Parnet P. Inactivation of the cerebral NFkappaB pathway inhibits interleukin-1beta-induced sickness behavior and c-Fos expression in various brain nuclei. Neuropsychopharmacology (2005) 30(8):1492–9. 10.1038/sj.npp.1300755 15900319

[45] Isung J Mobarrez F Nordstrom P Asberg M Jokinen J. Low plasma vascular endothelial growth factor (VEGF) associated with completed suicide. World J Biol Psychiatry (2012) 13(6):468–73. 10.3109/15622975.2011.624549 22098148

[46] Hilakivi-Clarke L. Role of estradiol in alcohol intake and alcohol-related behaviors. J Stud Alcohol (1996) 57(2):162–70. 10.15288/jsa.1996.57.162 8683965

[47] Okada M Hayashi N Kometani M Nakao K Inukai T. Influences of ovariectomy and continuous replacement of 17beta-estradiol on the tail skin temperature and behavior in the forced swimming test in rats. Jpn J Pharmacol (1997) 73(1):93–6. 10.1254/jjp.73.93 9032138

[48] Rachman IM Unnerstall JR Pfaff DW Cohen RS. Estrogen alters behavior and forebrain c-fos expression in ovariectomized rats subjected to the forced swim test. Proc Natl Acad Sci U S A (1998) 95(23):13941–6. 10.1073/pnas.95.23.13941 9811905 PMC24977

[49] Estrada-Camarena E Fernandez-Guasti A Lopez-Rubalcava C. Antidepressant-like effect of different estrogenic compounds in the forced swimming test. Neuropsychopharmacology (2003) 28(5):830–8. 10.1038/sj.npp.1300097 12637949

[50] Frye CA Wawrzycki J. Effect of prenatal stress and gonadal hormone condition on depressive behaviors of female and male rats. Horm Behav (2003) 44(4):319–26. 10.1016/S0018-506X(03)00159-4 14613726

[51] Walf AA Rhodes ME Frye CA. Antidepressant effects of ERbeta-selective estrogen receptor modulators in the forced swim test. Pharmacol Biochem Behav (2004) 78(3):523–9. 10.1016/j.pbb.2004.03.023 15251261

[52] Kessler RC Berglund P Demler O Jin R Koretz D Merikangas KR The epidemiology of major depressive disorder: results from the National Comorbidity Survey Replication (NCS-R). JAMA (2003) 289(23):3095–105. 10.1001/jama.289.23.3095 12813115

[53] Marcus SM Kerber KB Rush AJ Wisniewski SR Nierenberg A Balasubramani GK Sex differences in depression symptoms in treatment-seeking adults: confirmatory analyses from the Sequenced Treatment Alternatives to Relieve Depression study. Compr Psychiatry (2008) 49(3):238–46. 10.1016/j.comppsych.2007.06.012 18396182 PMC2759282

[54] Diaz-Veliz G Soto V Dussaubat N Mora S. Influence of the estrous cycle, ovariectomy and estradiol replacement upon the acquisition of conditioned avoidance responses in rats. Physiol Behav (1989) 46(3):397–401. 10.1016/0031-9384(89)90010-3 2623060

[55] Picazo O Estrada-Camarena E Hernandez-Aragon A. Influence of the post-ovariectomy time frame on the experimental anxiety and the behavioural actions of some anxiolytic agents. Eur J Pharmacol (2006) 530(1–2):88–94. 10.1016/j.ejphar.2005.11.024 16356491

[56] Pandaranandaka J Poonyachoti S Kalandakanond-Thongsong S. Differential effects of exogenous and endogenous estrogen on anxiety as measured by elevated T-maze in relation to the serotonergic system. Behav Brain Res (2009) 198(1):142–8. 10.1016/j.bbr.2008.10.043 19046994

[57] Diaz-Veliz G Urresta F Dussaubat N Mora S. Effects of estradiol replacement in ovariectomized rats on conditioned avoidance responses and other behaviors. Physiol Behav (1991) 50(1):61–5. 10.1016/0031-9384(91)90498-D 1946732

[58] Mora S Dussaubat N Diaz-Veliz G. Effects of the estrous cycle and ovarian hormones on behavioral indices of anxiety in female rats. Psychoneuroendocrinology (1996) 21(7):609–20. 10.1016/S0306-4530(96)00015-7 9044444

[59] Diaz-Veliz G Alarcon T Espinoza C Dussaubat N Mora S Ketanserin and anxiety levels: influence of gender, estrous cycle, ovariectomy and ovarian hormones in female rats. Pharmacol Biochem Behav (1997) 58(3):637–42. 10.1016/S0091-3057(97)90004-6 9329052

[60] Diaz-Veliz G Dussaubat N Mora S Ketanserin effects on rat behavioral responses: modifications by the estrous cycle, ovariectomy and estradiol replacement. Pharmacol Biochem Behav (1997) 57(4):687–92. 10.1016/S0091-3057(96)00394-2 9258995

[61] Pandaranandaka J Poonyachoti S Kalandakanond-Thongsong S. Anxiolytic property of estrogen related to the changes of the monoamine levels in various brain regions of ovariectomized rats. Physiol Behav (2006) 87(4):828–35. 10.1016/j.physbeh.2006.02.002 16545402

[62] Olivera-Lopez JI Molina-Hernandez M Tellez-Alcantara NP Jaramillo MT. Estradiol and neuropeptide Y (intra-lateral septal) reduce anxiety-like behavior in two animal models of anxiety. Peptides (2008) 29(8):1396–403. 10.1016/j.peptides.2008.04.002 18499302

[63] Milner TA McEwen BS Hayashi S Li CJ Reagan LP Alves SE. Ultrastructural evidence that hippocampal alpha estrogen receptors are located at extranuclear sites. J Comp Neurol (2001) 429(3):355–71. 10.1002/1096-9861(20010115)429:3<355::AID-CNE1>3.0.CO;2-# 11116225

[64] Milner TA Ayoola K Drake CT Herrick SP Tabori NE McEwen BS Ultrastructural localization of estrogen receptor beta immunoreactivity in the rat hippocampal formation. J Comp Neurol (2005) 491(2):81–95. 10.1002/cne.20724 16127691

[65] Lund TD Rovis T Chung WC Handa RJ. Novel actions of estrogen receptor-beta on anxiety-related behaviors. Endocrinology (2005) 146(2):797–807. 10.1210/en.2004-1158 15514081

[66] Autism Spectrum Disorders Working Group of the Psychiatric Genomics Consortium. Meta-analysis of GWAS of over 16,000 individuals with autism spectrum disorder highlights a novel locus at 10q24.32 and a significant overlap with schizophrenia. Mol Autism (2017) 8:21 10.1186/s13229-017-0137-9 28540026 PMC5441062

[67] Cronin S Tomik B Bradley DG Slowik A Hardiman O. Screening for replication of genome-wide SNP associations in sporadic ALS. Eur J Hum Genet (2009) 17(2):213–8. 10.1038/ejhg.2008.194 18987618 PMC2986065

[68] Smith EN Bloss CS Badner JA Barrett T Belmonte PL Berrettini W Genome-wide association study of bipolar disorder in European American and African American individuals. Mol Psychiatry (2009) 14(8):755–63. 10.1038/mp.2009.43 19488044 PMC3035981

[69] Dahl RE Gunnar MR. Heightened stress responsiveness and emotional reactivity during pubertal maturation: implications for psychopathology. Dev Psychopathol (2009) 21(1):1–6. 10.1017/S0954579409000017 19144219

[70] Romeo RD. The teenage brain: the stress response and the adolescent brain. Curr Dir Psychol Sci (2013) 22(2):140–5. 10.1177/0963721413475445 25541572 PMC4274618

[71] McCormick CM Mathews IZ. HPA function in adolescence: role of sex hormones in its regulation and the enduring consequences of exposure to stressors. Pharmacol Biochem Behav (2007) 86(2):220–33. 10.1016/j.pbb.2006.07.012 16901532

[72] Romeo RD Adolescence: a central event in shaping stress reactivity. Dev Psychobiol (2010) 52(3):244–53. 10.1002/dev.20437 20175102

[73] Romeo RD Pubertal maturation and programming of hypothalamic-pituitary-adrenal reactivity. Front Neuroendocrinol (2010) 31(2):232–40. 10.1016/j.yfrne.2010.02.004 20193707

[74] Lee PR Brady D Koenig JI. Corticosterone alters N-methyl-D-aspartate receptor subunit mRNA expression before puberty. Brain Res Mol Brain Res (2003) 115(1):55–62. 10.1016/S0169-328X(03)00180-3 12824055

[75] Giedd JN Rapoport JL. Structural MRI of pediatric brain development: what have we learned and where are we going? Neuron (2010) 67(5):728–34. 10.1016/j.neuron.2010.08.040 20826305 PMC3285464

