# Supplementary figures and images for: Prepubertal Ovariectomy Exaggerates Adult Affective Behaviors and Alters the Hippocampal Transcriptome in a Genetic Rat Model of Depression
Source: Front Endocrinol (Lausanne). 2018 Jan 22;8:373. doi: 10.3389/fendo.2017.00373 (PMC5786888; doi:10.3389/fendo.2017.00373)

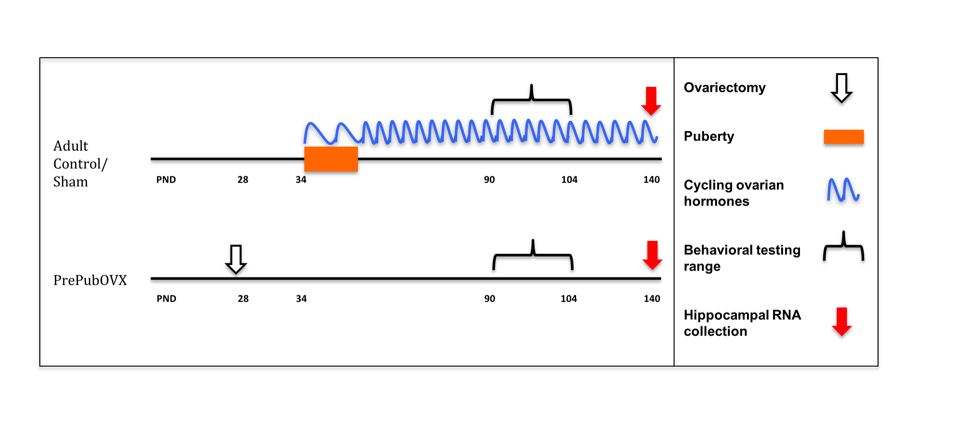

Supplement: Figure S1 — Schematics of the experimental design. Timings of prepubertal ovariectomy, behavioral testing, and hippocampal tissue collection are indicated. Puberty was marked to begin at postnatal day 34, by which time 20% of females demonstrate the criteria of puberty as described previously (Holehan AM, Merry BJ, The control of puberty in the dietary restricted female rat. Mech Ageing Dev (1985) 32:179–91.). [file image_1.png]

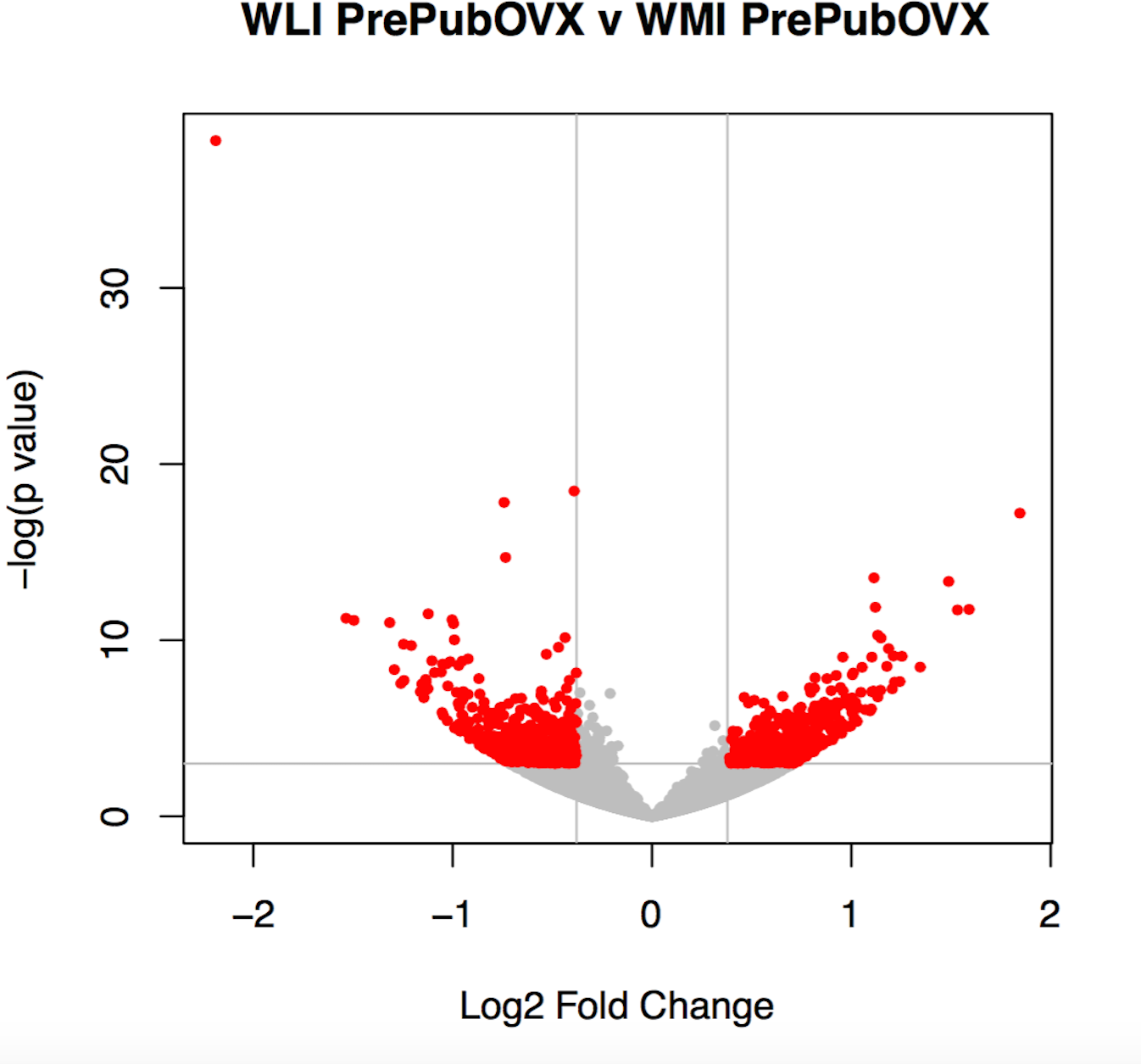

Supplement: Figure S2 — Differential RNA expression between Wistar Kyoto (WKY) less immobile (WLI) PrePubOVX and WKY more immobile (WMI) PrePubOVX hippocampus. Volcano plots showing −log (p-value) vs. log 2 fold change (FC) in RNA abundance in transcripts between the strains after PrePubOVX. Red circles denote significant differences (FDR corrected p < 0.05). FC cutoff is set at fold change (FC) > 1.3 or fold change < 0.7. [file image_2.tiff]

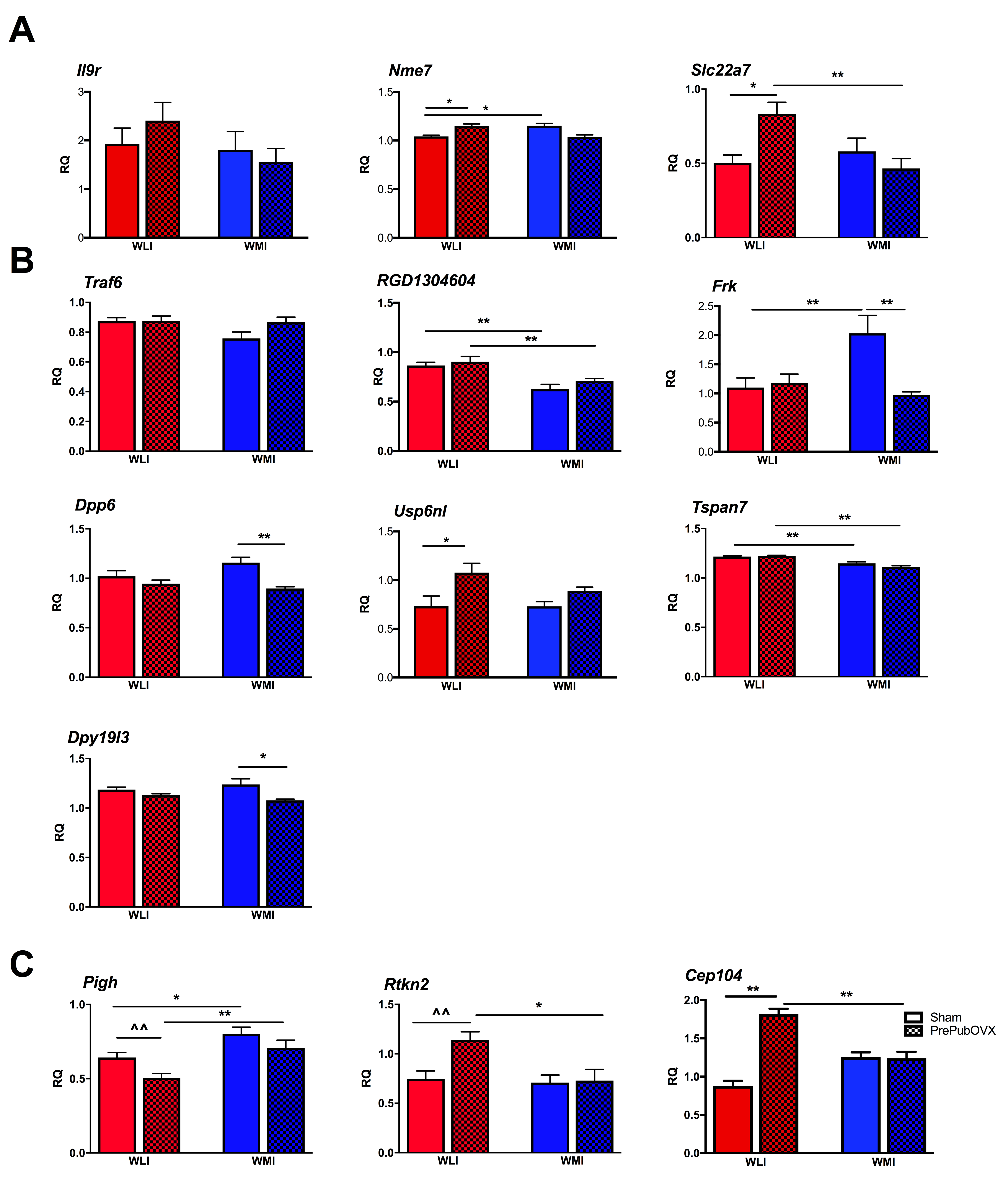

Supplement: Figure S3 — Quantitative RT-PCR analyses of differentially expressed genes (DEGs) selected to parallel behaviors in the forced swim test (FST) or open field test (OFT) (A) Wistar Kyoto (WKY) less immobile (WLI) Sham vs. WKY More immobile (WMI) Sham and WLI PrePubOVX vs. WLI Sham comparison, proposed to parallel WLI behavior in the FST: Il9r; Nme7; and Slc22a7. (B) WMI PrePubOVX vs. WMI Sham comparison, proposed to mirror their behavior in the OFT: Traf6, RGD1304604, Frk, Dpp6, Usp6nl, Tspan7, and Dpy19l3. (C) WLI PrePubOVX vs. WMI PrePubOVX comparison, with non-significant behavioral differences in the FST: Pigh, Rtkn2, and Cep104. Relative quantification (RQ) calculated using the 2−ΔΔCt method, after normalizing to GAPDH and a general hippocampal calibrator sample. **p < 0.01 and *p < 0.05 by Bonferroni-corrected post hoc analyses following a two-way ANOVA; ^^p < 0.01 hypothesis testing by Student’s t-test when ANOVA is significant or a trend (p < 0.1). Data are presented as mean ± SEM. [file image_3.tiff]

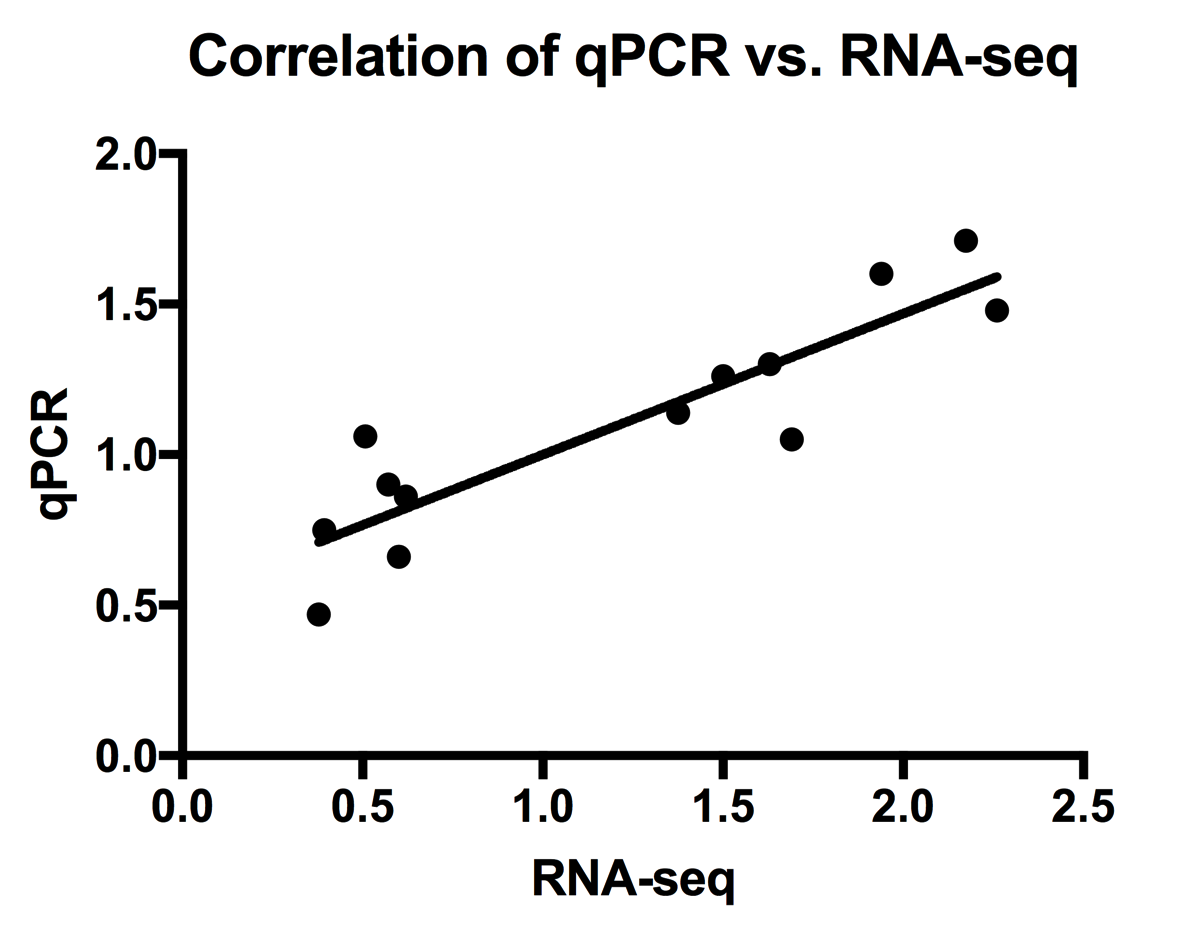

Supplement: Figure S4 — Significant Spearman correlation between RNA-sequencing (RNA-Seq) and RT-qPCR. Fold change determined by quantitative RT-PCR or RNA-Seq of genes shown on Figure S3 in Supplementary Material. [file image_4.tiff]

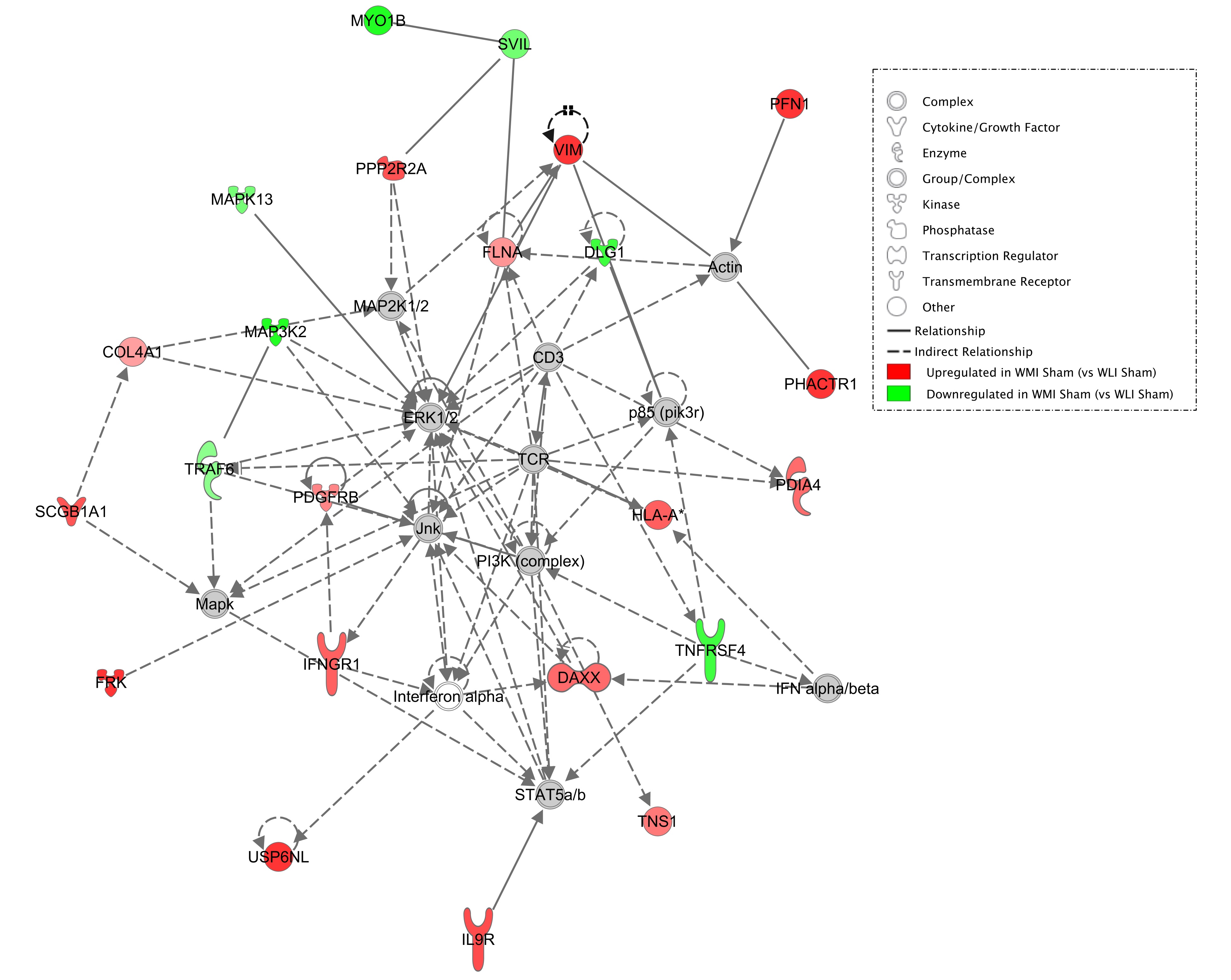

Supplement: Figure S5 — The most significant IPA Generated Network of Wistar Kyoto (WKY) more immobile (WMI) Sham vs. WKY less immobile (WLI) Sham differentially expressed genes (DEGs). DEGs with p < 0.01 were submitted to IPA with their corresponding fold changes using settings that allow for direct and indirect connections to other members of the network. Colored members are DEGs, while grey color indicates non-DEG connectors. The molecular/biological characteristics of the members are indicated in the legend. [file image_5.jpeg]

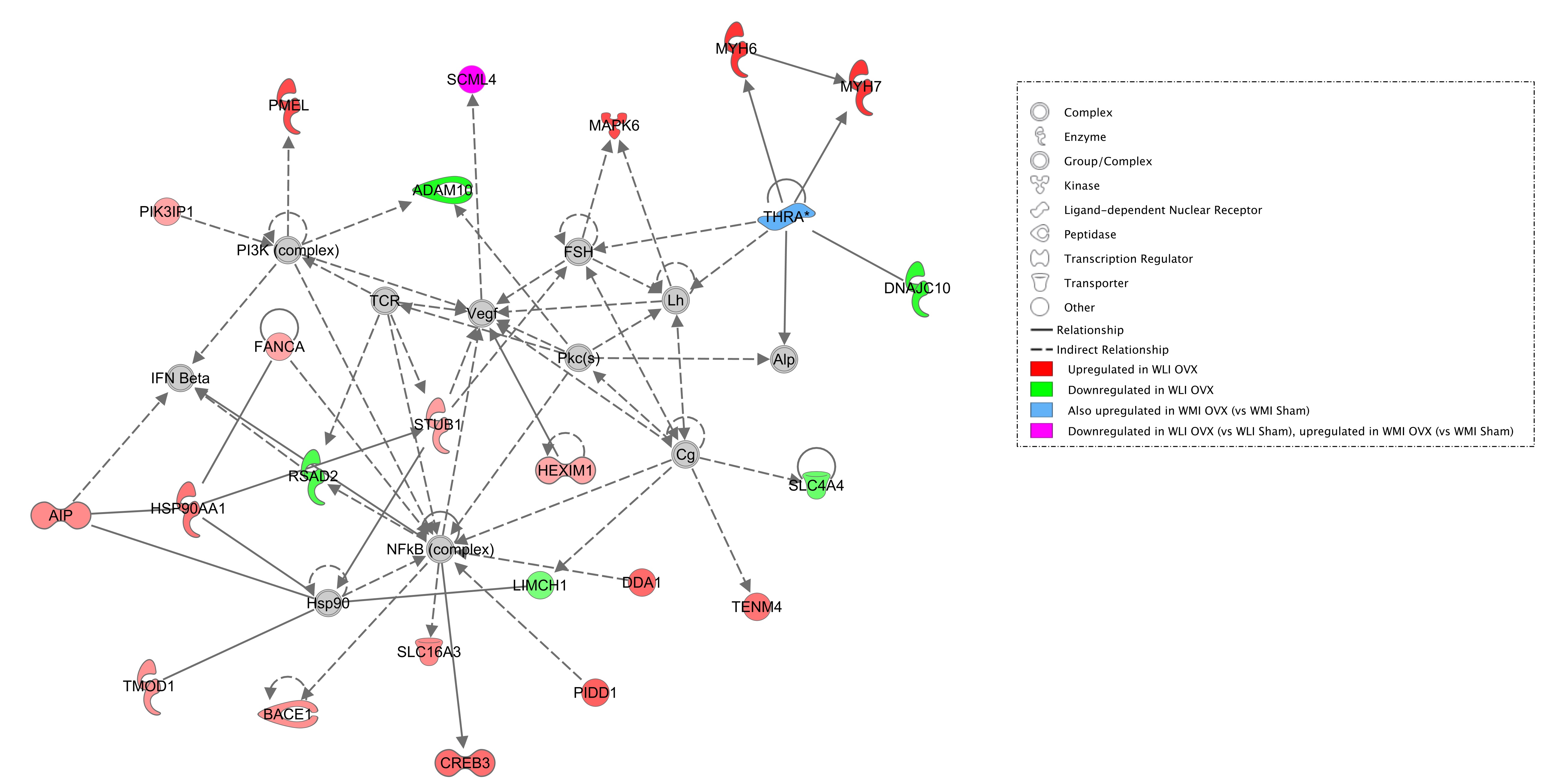

Supplement: Figure S6 — The most significant IPA Generated Network Wistar Kyoto (WKY) less immobile (WLI) Sham vs. WLI PrePubOVX differentially expressed genes (DEGs). DEGs with p < 0.01 were submitted to IPA with their corresponding fold changes using settings that allow for direct and indirect connections to other members of the network. Colored members are DEGs, while grey color indicates non-DEG connectors. The molecular/biological characteristics of the members are indicated in the legend. [file image_6.jpeg]

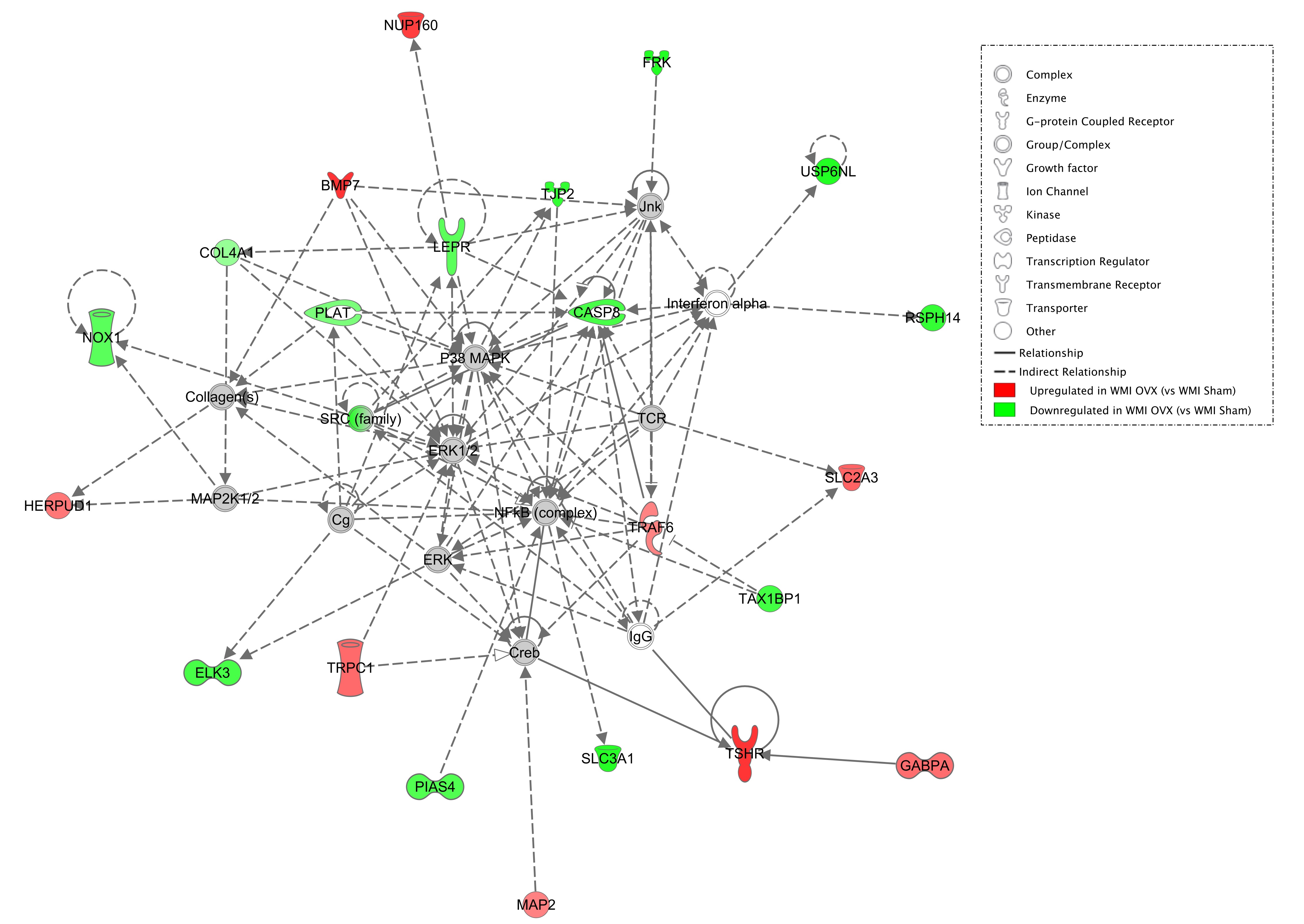

Supplement: Figure S7 — The most significant IPA Generated Network Wistar Kyoto (WKY) more immobile (WMI) Sham vs. WMI PrePubOVX differentially expressed genes (DEGs). DEGs with p < 0.01 were submitted to IPA with their corresponding fold changes using settings that allow for direct and indirect connections to other members of the network. Colored members are DEGs, while grey color indicates non-DEG connectors. The molecular/biological characteristics of the members are indicated in the legend. [file image_7.jpeg]
