# Supplementary material for: Prepubertal Ovariectomy Exaggerates Adult Affective Behaviors and Alters the Hippocampal Transcriptome in a Genetic Rat Model of Depression
Source: Front Endocrinol (Lausanne). 2018 Jan 22;8:373. doi: 10.3389/fendo.2017.00373 (PMC5786888; doi:10.3389/fendo.2017.00373)
Supplement: Supplementary file 8 [file table_1.doc]

**Supplemental Table 1. Quantitative RT-PCR primer sequences. F- Forward; R- Reverse**

| **Gene** |  | **Sequence 5’ - 3’** | **Amplicon length (bp)** |
| --- | --- | --- | --- |
| *Nme7* | *F* | CCTACGAAGACGTTCCGAGAA | 75 |
|  | *R* | GAGGGTCTCAGGTCGTAAATGC |  |
| *Il9r* | *F* | GCCCATCTTTCTTCTGCTGACT | 70 |
|  | *R* | GATTCTCTTCACCTTGGGAGACA |  |
| *Cep104* | *F* | AACAGCAGATGGCTCGCTAC | 87 |
|  | *R* | GTCTCTGCATCTCTGGCTCG |  |
| *Dpp6* | *F* | GCAAAGTGGATAAGCGATAAGGA | 73 |
|  | *R* | TCTCAACGTTCCGCAGTATGAC |  |
| *Dpy19l3* | *F* | TTGGACTTCTAACATCCGTGTACCT | 83 |
|  | *R* | TTCTCGCTCTACTTCCTTTATATTGGA |  |
| *Frk* | *F* | GCCTTACAGCGGGATGACA | 78 |
|  | *R* | CAGTTAGATGGCTGTGGTAGTCTGTAG |  |
| *Gapdh* | *F* | CAACTCCCTCAAGATTGTCAGCAA | 118 |
|  | *R* | GGCATGGACTGTGGTCATGA |  |
| *Usp6nl* | *F* | GAACCTGGTAAGGAGGATGAATACC | 97 |
|  | *R* | TGCCAACACTTCTTTGTCCATT |  |
| *Pigh* | *F* | TGTGCGAGAATAGCATGATCCT | 62 |
|  | *R* | ACCAAGCAGGCCTAAGATAGTGA |  |
| *RGD1304604* | *F* | TGCAGAAGCTGGACTACATGGT | 96 |
|  | *R* | CGAACACTTGCTGCGATTTC |  |
| *Rtkn2* | *F* | CCGCTTTGATCTCAGCATTG | 62 |
|  | *R* | TTTGTATTATGTCCGTCACGCTTT |  |
| *Traf6* | *F* | CGAAAAGATGCAGAGGAATCACT | 72 |
|  | *R* | GCCAACAGTCTCATGTGCAACT |  |
| *Slc22a7* | *F* | CCTTGGAGACTAAGTCATGGATCA | 99 |
|  | *R* | ACAACTCGGACGTGAACAGGTA |  |
| *Tspan7* | *F* | CATGAGATCAAGGACACCTTCCT | 71 |
|  | *R* | CTTTCATCTTTGCCGTTGTAGTTC |  |
